# Regulatory Variant rs2535629 in *ITIH3* Intron Confers Schizophrenia Risk By Regulating CTCF Binding and *SFMBT1* Expression

**DOI:** 10.1002/advs.202104786

**Published:** 2022-01-02

**Authors:** Yifan Li, Changguo Ma, Shiwu Li, Junyang Wang, Wenqiang Li, Yongfeng Yang, Xiaoyan Li, Jiewei Liu, Jinfeng Yang, Yixing Liu, Kaiqin Li, Jiao Li, Di Huang, Rui Chen, Luxian Lv, Xiao Xiao, Ming Li, Xiong‐Jian Luo

**Affiliations:** ^1^ Key Laboratory of Animal Models and Human Disease Mechanisms of the Chinese Academy of Sciences & Yunnan Province Kunming Institute of Zoology Chinese Academy of Sciences Kunming Yunnan 650204 China; ^2^ Kunming College of Life Science University of Chinese Academy of Sciences Kunming Yunnan 650204 China; ^3^ Yunnan Key Laboratory for Basic Research on Bone and Joint Diseases & Yunnan Stem Cell Translational Research Center Kunming University Kunming Yunnan 650214 China; ^4^ Henan Mental Hospital The Second Affiliated Hospital of Xinxiang Medical University Xinxiang Henan 453002 China; ^5^ Henan Key Lab of Biological Psychiatry International Joint Research Laboratory for Psychiatry and Neuroscience of Henan Xinxiang Medical University Xinxiang Henan 453002 China; ^6^ Key Laboratory of Intelligent Computing and Signal Processing of Ministry of Education Institutes of Physical Science and Information Technology Anhui University Hefei Anhui 230601 China; ^7^ Center for Excellence in Animal Evolution and Genetics Chinese Academy of Sciences Kunming Yunnan 650204 China; ^8^ KIZ‐CUHK Joint Laboratory of Bioresources and Molecular Research in Common Diseases Kunming Institute of Zoology Chinese Academy of Sciences Kunming Yunnan 650204 China

**Keywords:** dendritic spine, genetics, rs2535629, schizophrenia, *SFMBT1*

## Abstract

Genome‐wide association studies have identified 3p21.1 as a robust risk locus for schizophrenia. However, the underlying molecular mechanisms remain elusive. Here a functional regulatory variant (rs2535629) is identified that disrupts CTCF binding at 3p21.1. It is confirmed that rs2535629 is also significantly associated with schizophrenia in Chinese population and the regulatory effect of rs2535629 is validated. Expression quantitative trait loci analysis indicates that rs2535629 is associated with the expression of three distal genes (*GLT8D1*, *SFMBT1*, and *NEK4*) in the human brain, and CRISPR‐Cas9‐mediated genome editing confirmed the regulatory effect of rs2535629 on *GLT8D1*, *SFMBT1*, and *NEK4*. Interestingly, differential expression analysis of *GLT8D1*, *SFMBT1*, and *NEK4* suggested that rs2535629 may confer schizophrenia risk by regulating *SFMBT1* expression. It is further demonstrated that *Sfmbt1* regulates neurodevelopment and dendritic spine density, two key pathological characteristics of schizophrenia. Transcriptome analysis also support the potential role of *Sfmbt1* in schizophrenia pathogenesis. The study identifies rs2535629 as a plausibly causal regulatory variant at the 3p21.1 risk locus and demonstrates the regulatory mechanism and biological effect of this functional variant, indicating that this functional variant confers schizophrenia risk by altering CTCF binding and regulating expression of *SFMBT1*, a distal gene which plays important roles in neurodevelopment and synaptic morphogenesis.

## Graphical abstract

The study identifies rs2535629 as a plausibly causal regulatory variant at the 3p21.1 schizophrenia risk locus and demonstrates the regulatory mechanisms and biological effects of this functional variant, suggesting that this functional variant confers schizophrenia risk by altering CTCF binding and regulating expression of *SFMBT1*, a distal gene which plays important role in schizophrenia by regulating neurodevelopment and synaptic morphogenesis.

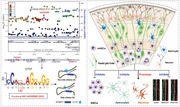

## 1 Introduction

Schizophrenia is a severe neuropsychiatric disorder that affects about 1% of the world's population.^[^ ^1^ ^]^ This disorder imposes an enormous economic and mental burden on patients, their families, and society.^[^ ^2^, ^3^ ^]^ Understanding the pathophysiology of schizophrenia is important as it can provide new therapeutic targets for drug development and treatment. Unfortunately, so far we know little about the pathogenesis of schizophrenia. The interactions between genetic and environmental factors are thought to be involved in schizophrenia. The heritability of schizophrenia was estimated as high as 80%,^[^ ^4^, ^5^ ^]^ indicating the dominant role of genetic components in this disorder. Lee et al. showed that single nucleotide polymorphisms (SNPs) captured ≈23% schizophrenia heritability (SNP heritability), indicating the pivotal role of common variants in schizophrenia.^[^ ^6^ ^]^ To identify common risk variants for schizophrenia, multiple genome‐wide association studies (GWASs) have been conducted in different continental populations and over 200 schizophrenia risk loci have been reported.^[^ ^7^, ^8^, ^9^, ^10^, ^11^, ^12^, ^13^, ^14^, ^15^ ^]^

Although GWASs have provided important insights into the genetic architecture of schizophrenia, challenges and obstacles remain in deciphering the underlying genetic mechanisms. For example, the lead (or index) variants reported by GWASs are usually not the causal variants. Genetic variants in linkage disequilibrium (LD) often show similar *P* values, impeding the identification of causal variants. Moreover, most of the risk SNPs identified by GWASs are located in non‐coding regions, suggesting that modulating gene expression is a primary manner that the risk variants exert their biological effects on schizophrenia susceptibility. Finally, gene regulation is a complex process and gene (or genes) nearest to the reported risk variants are not necessarily the target genes of the risk variants (risk variants may confer disease risk by modulating distal gene).^[^ ^16^, ^17^, ^18^, ^19^, ^20^, ^21^ ^]^ To pinpoint the plausible causal variants, we used the functional genomics approach to identify the schizophrenia risk variants that affected binding of transcription factors (TFs) recently.^[^ ^22^ ^]^ By integrating chromatin immunoprecipitation followed by sequencing (ChIP‐seq) and DNA binding motifs (position weight matrix, PWM), we identified 132 TF binding‐disrupting SNPs from the reported schizophrenia risk loci (Figure S1, Supporting Information).^[^ ^22^ ^]^ Of note, a CTCF binding‐disrupting functional variant (rs2535629, located on 3p21.2) showed robust association with schizophrenia (*P* = 9.85 × 10^−13^)^[^ ^23^ ^]^ and bipolar disorder (*P* = 4.93 × 10^−7^).^[^ ^24^ ^]^ More importantly, we conducted an independent genetic association study in Chinese population (N = 4291 cases and 7847 controls) to replicate the association between rs2535629 and schizophrenia, and the result showed that rs2535629 was robustly associated with schizophrenia in Chinese population (*P* = 1.86 × 10^−9^), with the same risk allele as in European populations (**Table** **1**). These lines of evidence suggest that rs2535629 is a potential causal variant with regulatory effect. However, the regulatory mechanisms and biological effects of rs2535629 in schizophrenia remain unclear. Functional understanding of this TF binding‐disrupting variant will not only provides important insights into the genetic etiology and biological mechanisms of schizophrenia, but also brings new opportunities to elucidate the pathogenesis of schizophrenia and potential therapeutic targets.

**Table 1:** rs2535629 is significantly associated with schizophrenia in Chinese, meta‐analysis of rs2535629 in Europeans and Chinese populations

SNP ID	Sample	Cases	Controls	Allele (A1/A2)	*P* value	OR^a)^
rs2535629	Chinese sample	4291	7847	A/G	1.86 × 10^−9^	0.8461
	PGC2+EAS	56418	78818	A/G	1.87 × 10^−9^	0.9466
	Combined	60709	86665	A/G	2.90 × 10^−14^	0.9364

^a)^ OR (odds ratio) was based on A1 allele.

In this study, we systematically investigated the regulatory mechanisms and biological effects of rs2535629. We first showed that rs2535629 is located in an active regulatory element bound with CTCF (in neuronal cells)^[^ ^22^ ^]^ and confirmed that CTCF could bind to the genomic sequence containing rs2535629. We then validated the regulatory effect of rs2535629 with reporter gene assays. Interestingly, although rs2535629 is located in the intron 7 of *ITIH3* gene, expression quantitative traits loci (eQTL) analysis showed that this functional SNP was associated with expression of three distal genes (*GLT8D1, NEK4*, and *SFMBT1*) in the human brain. CRISPR‐Cas9‐mediated genome editing further validated the regulatory effect of rs2535629 on *GLT8D1, NEK4*, and *SFMBT1*. Of note, gene expression showed that *SFMBT1* was dysregulated in neurons induced from fibroblasts of schizophrenia cases compared with controls, and rs2535629 physically interacts with *SFMBT1*, supporting that this functional variant confers risk of schizophrenia by modulating *SFMBT1* expression. Finally, we explored the potential roles of *Sfmbt1* in schizophrenia pathogenesis by using the neural stem cell model (including proliferation and differentiation of neural stem cells) and dendritic spine density analysis. Our study identified rs2535629 as a likely causal variant (for schizophrenia) at the 3p21.2 risk locus and demonstrated that this functional variant regulates *SFMBT1* expression by affecting CTCF binding, offering pivotal insights into genetic mechanisms and pathogenesis of schizophrenia.

## 2 Results

### 2.1 Functional Genomics Identified rs2535629 as a Plausible Causal Variant at the 3p21.2 Locus

**Figure 1:**
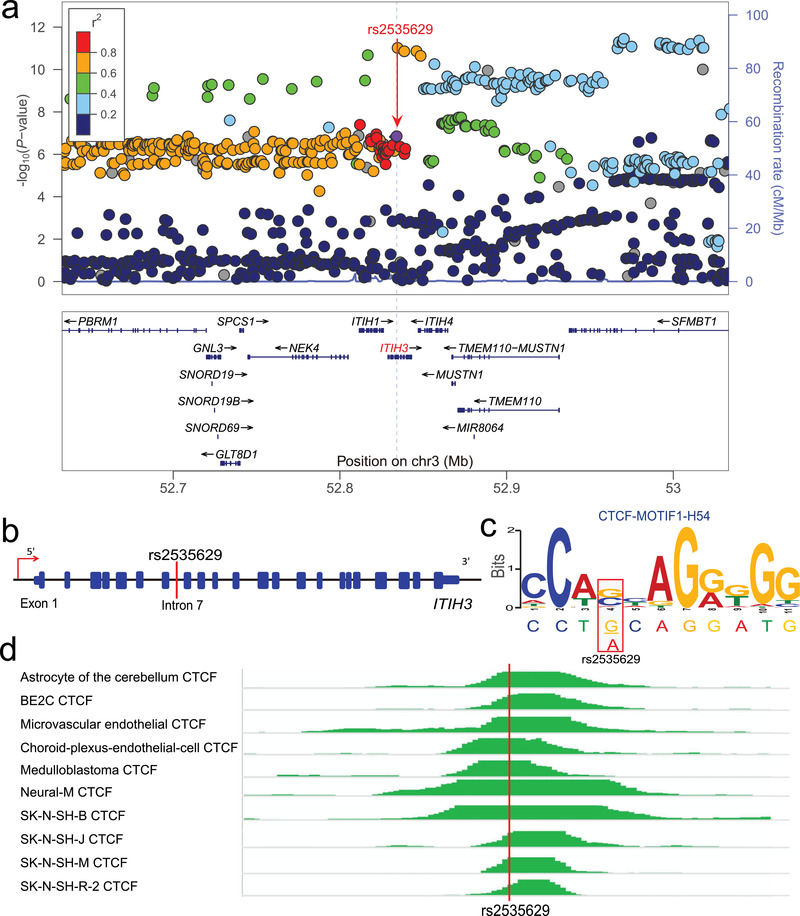
rs2535629 is a functional variant that disrupts CTCF binding at the 3p21.1 risk locus. a) Locuszoom plot showed that rs2535629 is located in a genomic region with multiple SNPs showing significant associations with schizophrenia.^[^ ^25^ ^]^ b) rs2535629 is located in the seventh intron of *ITIH3*. c) rs2535629 is located in the binding motif of CTCF and different alleles of rs2535628 altered CTCF binding. d) Chromatin immunoprecipitation and sequencing (ChIP‐Seq) data showed that CTCF preferentially bound to genomic sequence containing rs2535629 in neuronal cells and tumor cell lines from the human brain (including neuroblastoma and medulloblastoma cell lines) in vivo. The heights of the colored graphs reflect the ChIP‐Seq signal intensities, and the location of rs2535629 is highlighted with the red line.

Our functional genomics analysis showed that rs2535629 is a functional variant at the 3p21.2 schizophrenia risk locus.^[^ ^22^ ^]^ SNP rs2535629 resides in a genomic region with multiple SNPs showing significant associations with schizophrenia (**Figure** **1**a). The association significance between rs2535629 and schizophrenia in Europeans (40 675 cases and 64 643 controls) is *P* = 1.48 × 10^−7^ (Figure 1a).^[^ ^25^ ^]^ However, rs2535629 showed robust association with schizophrenia (*P* = 9.85 × 10^−13^) in a recent larger GWAS (67 390 cases and 94 015 controls).^[^ ^23^ ^]^ If rs2535629 is a bona fide causal SNP, it may also be associated with schizophrenia in non‐European populations such as the Chinese population. To further verify the association between rs2535629 and schizophrenia, we conducted a replication study in Chinese population (4291 cases and 7847 controls). We found that rs2535629 was also significantly associated with schizophrenia in the Chinese cohort (*P* = 1.86 × 10^−9^) (Table 1), with the risk allele as reported in the Europeans. We further investigated the association between rs2535629 and schizophrenia by combining results from European GWAS (56 418 cases and 78 818 controls)^[^ ^9^ ^]^ and our cohort. Meta‐analysis (a total of 60 709 cases and 86 665 controls) showed a strong association between rs2535629 and schizophrenia (*P* = 2.90 × 10^−14^) (Table 1). Of note, rs2535629 has a similar minor allele frequency (MAF) in European (MAF, 37%, minor allele: A) and southern Han Chinese (MAF, 37%) populations (Figure S2, Supporting Information). In northern Han Chinese (Han Chinese in Beijing, CHB), the MAF is 47%. In line with the similar MAF in European and Chinese populations, LD analysis also showed a similar LD structure of the genomic region surrounding rs2535629 in European (EUR) and Chinese populations (Figure S3, Supporting Information), supporting that the genomic region surrounding rs2535629 may be a common risk locus for schizophrenia. rs2535629 lies in the seventh intron of *ITIH3* gene on chromosome 3 (Figure 1b). Our previous functional genomics showed that rs2535629 is located in a well‐defined CTCF binding motif, with different alleles of rs2535629 affected CTCF binding affinities (Figure 1c). The strong CTCF ChIP‐Seq signal covering rs2535629 indicated that CTCF bound to the genomic region containing rs2535629 in neuronal cells and tumor cell lines from the human brain (including neuroblastoma and medulloblastoma cell lines) in vivo (Figure 1d).

### 2.2 Reporter Gene Assays Validated rs2535629 as a Regulatory Variant

**Figure 2:**
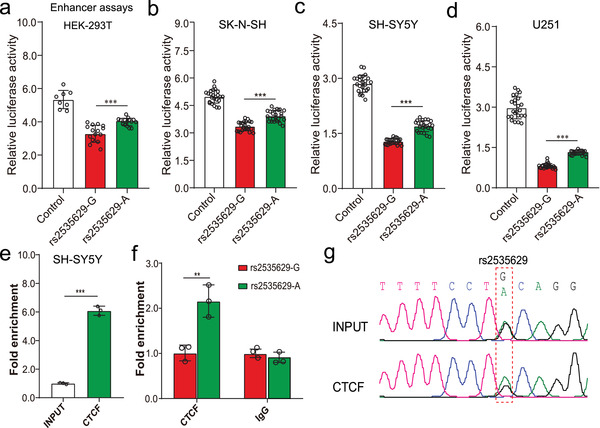
Validation of the regulatory effects of rs2535629. a–d) Dual‐luciferase reporter gene assays (enhancer assay, the cloned fragments were cloned into pGL3‐promoter vector, which is used to test the enhancer activity of the cloned fragments) showed that the G allele (risk allele) of rs2535629 conferred significant lower luciferase activity than A allele in HEK‐293T cells (a), SK‐N‐SH cells (b), SH‐SY5Y cells (c), and U251 cells (d). Data represent mean ± SD, n = 8 for control group, n = 16 for each experimental group in HEK293T, and n = 24 for each group in SK‐N‐SH, SH‐SY5Y, and U251 cells. e) Significant enrichment of CTCF on genomic sequence containing rs2535629. DNA templates from cross‐linked chromatins of cells (without CTCF immunoprecipitation) were used in INPUT group, and DNA templates from cross‐linked chromatins of cells (with CTCF immunoprecipitation) were used in CTCF group. n = 3 for each group. f) ChIP‐AS‐qPCR revealed that A allele of rs2535629 was preferentially bound to CTCF compared with G allele. DNA templates from cross‐linked chromatins of cells (with CTCF immunoprecipitation (left panel) and IgG immunoprecipitation (left panel)) were used. Different alleles of rs2535629 had different CTCF enrichment efficiencies and the results reflected that the binding affinity (to CTCF) of A allele was higher than G. n = 3 for each group. g) ChIP‐Sanger sequencing showed stronger CTCF binding to A allele of rs2535629 than G allele. *P* values were calculated using the Student's t‐test (two‐tailed) in (a–f). **P <0.01, ***P < 0.001.

As rs2535629 resides in the intronic region, we hypothesized that this TF binding‐disrupting SNP may be located in a regulatory element to modulate its target gene (or genes). To test the regulatory activity of the genomic sequence containing rs2535629, we conducted dual‐luciferase reporter gene assays. Reporter gene assays showed that rs2535629 is located in a repressive element, as the luciferase activity of the fragments containing rs2535629 was lower than controls (**Figure** **2**a–d). In addition, reporter gene assays also revealed that different alleles of rs2535629 affected luciferase activity significantly (Figure 2a–d). The G allele (risk allele) of rs2535629 conferred significantly lower luciferase activity than A allele in all the tested cells (Figure 2a–d). These results validated the regulatory effect (or functional consequences) of rs2535629 and suggested that rs2535629 may exert its biological effect on schizophrenia by modulating the transcription activity of the regulatory element it located.

### 2.3 rs2535629 Affects CTCF Binding

To further verify if different alleles of rs2535629 alter CTCF binding affinity, we conducted ChIP‐Allele‐Specific‐qPCR (ChIP‐AS‐qPCR) in SH‐SY5Y (a neuroblastoma cell line) cells. We first carried out ChIP‐qPCR and found significant enrichment of CTCF binding on the sequence containing rs2535629 compared with the control, supporting that CTCF preferentially binds to the genomic region containing rs2535629 (Figure 2e). ChIP‐AS‐qPCR further revealed that CTCF preferentially binds to the A allele of rs2535629 compared with G allele (Figure 2f). Consistent with ChIP‐AS‐qPCR results, ChIP‐Sanger sequencing also indicated that CTCF prefers to bind the A allele than G allele at rs2535629 (Figure 2g). These results demonstrated that CTCF bound to the genomic sequence containing rs2535629 in vivo and revealed that different alleles of rs2535629 altered the binding affinity of CTCF.

### 2.4 rs2535629 is Associated with *SFMBT1* Expression in the Human Brain

**Figure 3:**
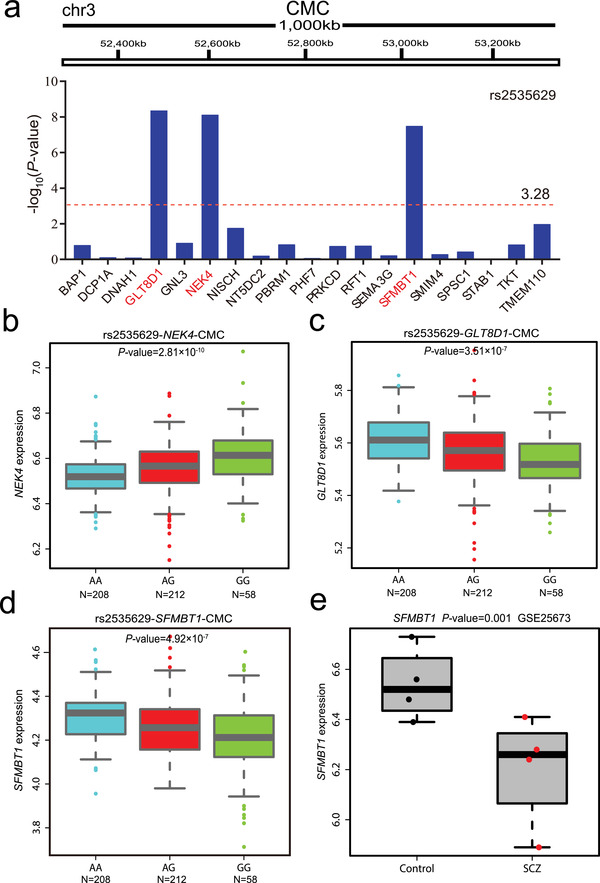
rs2535629 is associated with the expression of *GLT8D1, NEK4*, and *SFMBT1* in human brain tissues. a) eQTL analysis of rs2535629 and its surrounding genes (within 1M bp window, 500 kb upstream, and 500 kb downstream of rs2535629, respectively) in CMC brain eQTL dataset. Expression of *GLT8D1, NEK4*, and *SFMBT1* showed the most significant associations with rs2535629. The horizontal red line shows the corrected *P* value (‐log_10_ (*P*value/number of genes)). b–d) eQTL analysis demonstrated the correlations between three rs2535629 genotypes and the expression of *NEK4, GLT8D1*, and *SFMBT1*. e) *SFMBT1* was significantly down‐regulated in neuron (induced from hiPSs) of schizophrenia cases compared with controls in GSE25673 dataset^[^ ^28^ ^]^ (*P* = 0.001).

As rs2535629 is located in the intronic region and is a regulatory variant, we hypothesized that this functional variant may confer schizophrenia risk by modulating gene expression. We therefore examined if rs2535629 is associated with gene expression in the human brain by using eQTL data from the Common Mind Consortium (CMC).^[^ ^26^ ^]^ eQTL analysis showed that rs2535629 was significantly associated with the expression of three genes (*GLT8D1, NEK4*, and *SFMBT1*) in the human brain (corrected *P* < 0.05, **Figure** **3**a). Although rs2535629 is located in intronic region of *ITIH3*, no significant association between rs2535629 and *ITIH3* expression was detected in the human brain. Of note, the risk allele (G) of rs2535629 is associated with a higher expression level of *NEK4* (Figure 3b), and a lower expression level of *SFMBT1* and *GLT8D1* (Figure 3c,d). These eQTL results indicated that rs2535629 may confer schizophrenia risk by regulating these three genes.

### 2.5 rs2535629 Regulates *SFMBT1* Expression via CTCF

**Figure 4:**
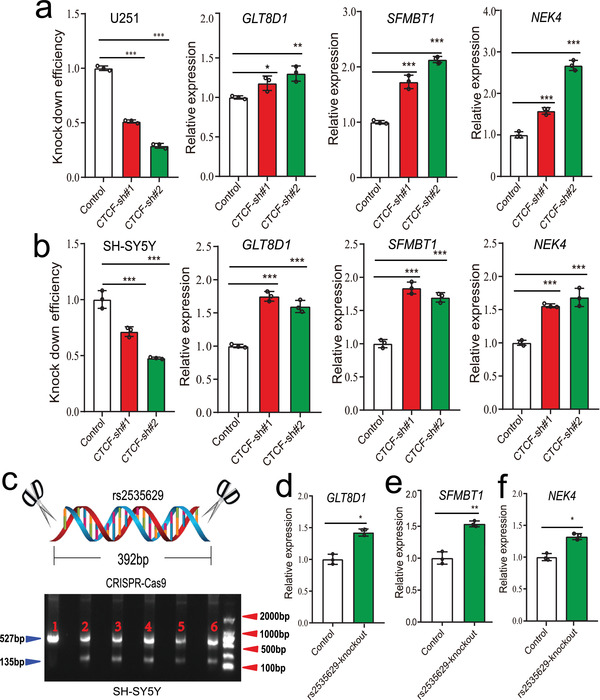
Regulatory effects of *CTCF* and rs2535629 on *GLT8D1, NEK4*, and *SFMBT1*. a) CTCF knockdown in U251 cells affected *GLT8D1*, *SFMBT1*, and *NEK4* expression significantly. b) CTCF knockdown in SH‐SY5Y cells up‐regulated expression of *GLT8D1*, *SFMBT1*, and *NEK4* significantly. c–f) rs2535629 knockout up‐regulated expression of *GLT8D1*, *SFMBT1*, and *NEK4* in SH‐SY5Y cells. (c) Schematic diagram of rs2535629 knockout. The fragment length of wildtype cells is 527 bp. However, the fragment length of the genomic sequence containing rs2535629 in knocked‐out cells is 135 bp (as a 392 bp sequence containing rs2535629 was deleted by CRISPR‐Cas9). n = 3 for each group. Two‐tailed Student's t‐test was used for detection of statistical significance, *P < 0.05, **P <0.01, ***P < 0.001.

To test if rs2535629 regulates the expression of its eQTL genes by interacting with CTCF, we knocked down *CTCF* expression in SH‐SY5Y (a neuroblastoma cell line) and U251 (a human glioblastoma cell line) cells (**Figure** **4**a,b). *CTCF* knockdown affected the expression of *NEK4, GLT8D1*, and *SFMBT1*, indicating that *CTCF* regulates these three genes (Figure 4a,b). We also examined the effect of CTCF knockdown on *Sfmbt1* expression in GEO dataset (GSE99230). In GSE99230 dataset, *CTCF* knocked‐down up‐regulated *Sfmbt1* expression by 25%, which is consistent with our observation. Of note, all three genes showed significant up‐regulation in *CTCF* knockdown cells, suggesting a repressive effect of *CTCF* on these three genes.

To further verify if the regulatory effects of CTCF on *NEK4, GLT8D1*, and *SFMBT1* are mediated by rs2535629, we knocked out a 392 bp genomic sequence containing rs2535629 in SH‐SY5Y cell line (Figure 4c). Knockout of the genomic region containing SNP rs2535629 up‐regulated the expression of *NEK4, GLT8D1*, and *SFMBT1* (Figure 4d–f), corroborating that the sequence containing rs2535629 acts as a repressive element for these genes (which was consistent with reporter gene assays). Considering that CTCF preferentially binds to the A allele rs2535629 and CTCF knockdown resulted in significant up‐regulation of *NEK4, GLT8D1*, and *SFMBT1*, these data collectively suggested that rs2535629 may regulate the expression of *NEK4, GLT8D1*, and *SFMBT1* via interacting with CTCF.

### 2.6 Dysregulation of *SFMBT1* in Schizophrenia Cases

To further test if rs2535629 conferred schizophrenia risk by modulating the expression of *NEK4*, *GLT8D1*, and *SFMBT1*, we examined the expression level of these three genes in brains of schizophrenia cases and controls using data from the PsychENCODE^[^ ^27^ ^]^ (559 cases and 936 controls). None of them showed significant expression alteration in brains of schizophrenia cases compared with controls. We thus further examined the expression of these three genes in neurons (differentiated from human induced pluripotent stem cells, hiPSCs) induced from hiPSCs of schizophrenia cases compared with controls (GSE25673).^[^ ^28^ ^]^ *GLT8D1* and *NEK4* did not show significant expression change in neurons induced from schizophrenia cases compared with controls. However, *SFMBT1* was significantly down‐regulated in neurons induced from schizophrenia cases compared with controls (*P* = 8.90 × 10^−4^, FDR<0.05) (Figure 3e). These data suggested that dysregulation of *SFMBT1* might have a role in schizophrenia. Of note, eQTL analysis predicted down‐regulation of *GLT8D1* and *SFMBT1* in schizophrenia cases compared with controls as the risk allele (G) was associated with lower expression of *GLT8D1* and *SFMBT1* (Figure 2c,d), which was concordant with the observation of significant down‐regulation of *SFMBT1* in neurons differentiated from hiPSCs of schizophrenia cases. Taken together, these data suggested that the functional regulatory variant rs2535629 might confer schizophrenia risk by regulating *SFMBT1*.

### 2.7 *Sfmbt1* Regulates Proliferation of NSCs

Our above data suggest that the gene (or genes) regulated by rs2535629 may be involved in the pathogenesis of schizophrenia. To test this hypothesis, we investigated the role of genes regulated by rs2535629 in neurodevelopment. As we have demonstrated the potential role of *GLT8D1* in schizophrenia in our previous study^[^ ^29^ ^]^ and expression analysis showed no significant change of *NEK4* and *GLT8D1* in schizophrenia cases compared with controls, we focused on *SFMBT1* in this study.

**Figure 5:**
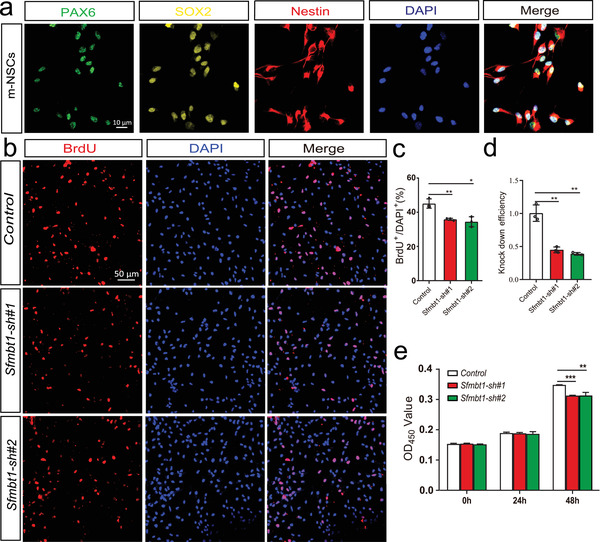
*Sfmbt1* knockdown affected proliferation of NSCs. a) Co‐labeling (Immunofluorescence) with three well‐characterized markers for NSCs validated the identity of isolated mouse neural stem cells. b,c) BrdU assays showed that *Sfmbt1* knockdown impaired the proliferation abilities of NSCs. n = 3 (3 independent biological replicates) for each group and five technical replicates were performed for each biological replicate. c) Quantification data for (b). d) Relative expression of *Sfmbt1* in control and shRNA‐mediated knocked‐down mNSCs (measured by real‐time qPCR) (n = 3). e) CCK8 assays revealed that *Sfmbt1* knockdown affected proliferation of NSCs significantly. n = 10 for each group. *P* values were calculated using the Student's t‐test (two‐tailed) in (c–e). *P < 0.05, **P <0.01, ***P < 0.001.

To date, the pathogenesis of schizophrenia remains largely unknown. However, accumulating evidence supports the neurodevelopmental hypothesis of schizophrenia (which posits that schizophrenia is mainly attributed to abnormal brain development). Consistent with the neurodevelopmental hypothesis, the neural stem cell model (a model that was widely used to explore the function of SCZ‐associated genes)^[^ ^30^, ^31^, ^32^, ^33^, ^34^ ^]^ also has revealed the pivotal of some schizophrenia risk genes in neurodevelopment (including proliferation, migration, and differentiation). We thus used the neural stem cell model to investigate the role of *SFMBT1* in neurodevelopment. Of note, 84% coding sequence (https://blast.ncbi.nlm.nih.gov/Blast) and 89% protein sequence (https://www.uniprot.org/align) of *SFMBT1* were identical between humans and mice, suggesting that the function of *SFMBT1* is relatively conserved in humans and mice. The isolated mouse neural stem cells (mNSCs) expressed the three well‐characterized markers for NSCs (SOX2, NESTIN, and PAX6) (**Figure** **5**a), indicating that these cells were NSCs. We then knocked‐down *Sfmbt1* expression in mNSCs using shRNAs (Figure 5). Both BrdU (5‐bromodeoxyuracil nucleotide) incorporation and CCK8 assays showed that *Sfmbt1* knock‐down impaired the proliferation of NSCs significantly (Figure 5b–d). These results demonstrated the important role of *Sfmbt1* in regulating the proliferation of NSCs.

### 2.8 *Sfmbt1* Regulates Differentiation of NSCs

**Figure 6:**
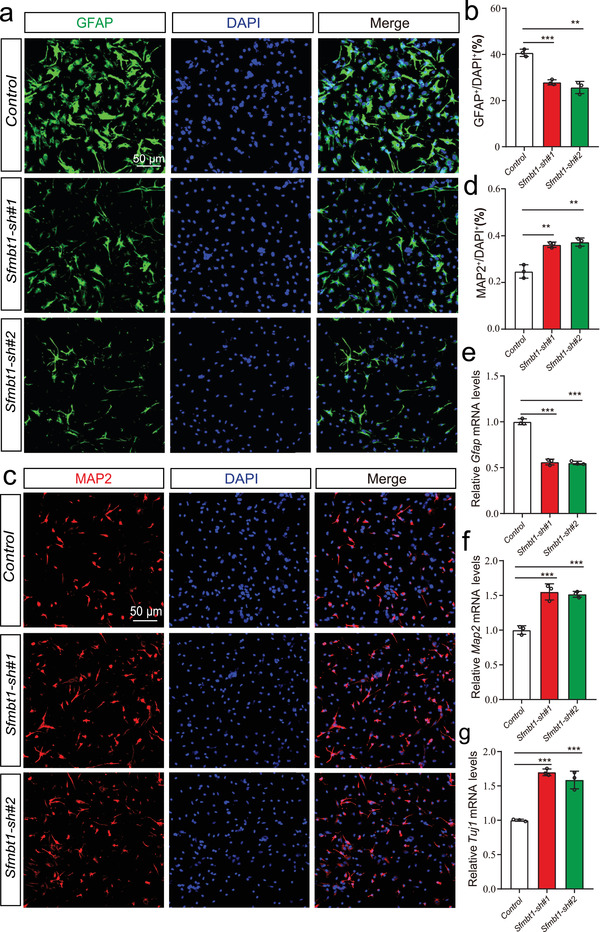
*Sfmbt1* knockdown affected differentiation of NSCs. a,b) *Sfmbt1* knockdown inhibited the differentiation of NSCs into GFAP positive astrocytes. n = 3 (3 independent biological replicates) for each group and four technical replicates were performed for each biological replicate. c,d) *Sfmbt1* knockdown promoted the differentiation of NSCs into MAP2 positive neurons. n = 3 (3 independent biological replicates) for each group and four technical replicates were performed for each biological replicate. e–g) qPCR showed the expression change of *Gfap*, *Map2*, and *Tuj1* in *Sfmbt1* knockdown cells. *P* values were calculated using the Student's t‐test (two‐tailed) in (b,d) and (e–g). **P <0.01, ***P < 0.001.

In addition to proliferation (which occurs during the early developmental stage), differentiation also plays an important role in neurodevelopment as functionally distinct neurons are generated in the process of differentiation. To further investigate the role of *Sfmbt1* in neuronal differentiation, we differentiated the mNSCs into neurons and glia cells. The results showed that the proportion of GFAP positive glia cells was significantly reduced in *Sfmbt1* knocked‐down cells compared with controls (**Figure** **6**a,b). However, *Sfmbt1* knock‐down resulted in a significant increase of MAP2 positive neurons (Figure 6c,d). We also examined the mRNA expression level of *Gfap* (a marker for glia cells), *Map2* (a marker for mature neurons), and *Tuj1* (a marker for newly generated immature post‐mitotic neurons) with qPCR. Consistent with immunofluorescence results, the mRNA expression level of *Gfap* was significantly decreased in *Sfmbt1* knocked‐down cells compared with controls (Figure 6e). By contrast, the mRNA expression level of *Map2* and *Tuj1* was significantly increased (Figure 6f,g). These results indicate that *Sfmbt1* plays a role in the differentiation of NSCs.

### 2.9 *Sfmbt1* Regulates Neurodevelopment‐Associated Pathways

**Figure 7:**
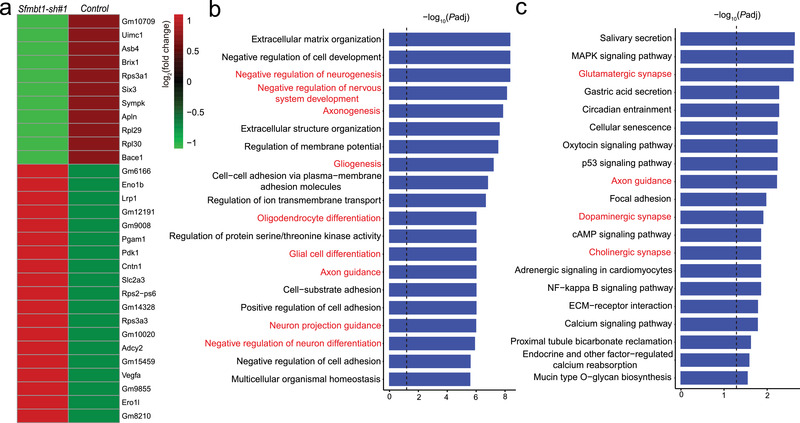
*Sfmbt1* regulates neurodevelopment‐associated pathways and synaptic organization. a) The top 30 genes that showed the most significant expression differences in *Sfmbt1* knocked‐down and control cells. b) GO analysis of the DEGs. The DEGs were enriched in schizophrenia‐associated pathways, including negative regulation of nervous system development, negative regulation of neurogenesis, axonogenesis, glial cell differentiation, and gliogenesis. The black vertical dashed bar indicates adjusted *P*<0.05. Neurodevelopment‐associated pathways were highlighted with red. c) KEGG analysis showed enrichment of DEGs in neuronal synapse (including Glutamatergic synapse, Dopaminergic synapse, MAPK signaling pathway, Cholinergic synapse, and Calcium signaling pathways) and signaling pathways, indicating the pivotal role of *Sfmbt1* in the development and function of synapse. The black vertical bar indicates adjusted *P*<0.05.

To further investigate the pathways (or biological processes) regulated by *Sfmbt1*, we conducted transcriptome analysis using RNA sequencing (RNA‐Seq). A total of 3092 genes showed significant expression change (*P*adj<0.01, |Log_2_ (fold change)|>0.5) in *Sfmbt1* knocked down NSCs compared with controls (Figure S4, Supporting Information). The top 30 differentially expressed genes (DEGs) are shown in **Figure** **7**a. Gene Ontology (GO) analysis showed that the DEGs were mainly enriched in neurodevelopment‐associated pathways, including negative regulation of nervous system development, negative regulation of neurogenesis, axonogenesis, glial cell differentiation, and gliogenesis (Figure 7b). Kyoto Encyclopedia of Genes and Genomes (KEGG) analysis showed that the DEGs were mainly enriched in the pathways related to the neuronal synapse (including glutamatergic, dopaminergic, and cholinergic) (Figure 7c), suggesting that *Sfmbt1* has an important function in the central nervous system (CNS) and synaptic transmission. In addition, DEGs were also enriched in cell cycle‐related pathways (Figure 7c) such as p53 signaling pathway, which is consistent with the results of BrdU and CCK8 proliferation assays. Of note, previous studies have shown that schizophrenia risk genes were enriched in pathways related to neurodevelopment and synaptic transmission.^[^ ^35^, ^36^, ^37^, ^38^ ^]^ Besides, spatio‐temporal expression pattern analysis showed that *SFMBT1* expression level was higher in the developing brain (prenatal stages) than adulthood brain (postnatal stages) (Figure S5, Supporting Information),^[^ ^39^ ^]^ suggesting this gene may have a pivotal role in human brain development. Collectively, these results indicate that *Sfmbt1* regulates important pathways related to neuronal‐related cell components and neurological diseases, further indicating the important role of *Sfmbt1* in the CNS.

### 2.10 *Sfmbt1* Knockdown Affects the Dendritic Spine Density of Neurons

**Figure 8:**
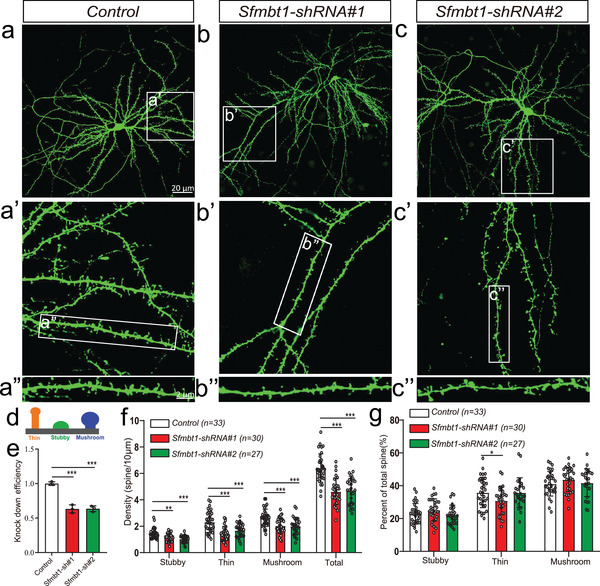
*Sfmbt1* knockdown affected the density and proportion of the dendritic spines. a–c) Confocal images of whole neurons transfected with control and *Sfmbt1* knockdown vectors (scale bars represent 20 µm). Dendritic branches were captured from each corresponding neuron, respectively (scale bars represent 2 µm). Green fluorescence is the result of immunofluorescence of GFP protein expressed by co‐transfected GFP vector. d) A schematic diagram to show different types of spines. e) Relative expression of *Sfmbt1* in control and *Sfmbt1* knocked‐down neurons (measured by real‐time qPCR). (n = 3). f) Density analysis of each dendritic spine subtype. g) Percentage analysis of each dendritic spine subtype. n = 33 for control group, n = 30 for shRNA#1 group, and n = 27 for shRNA#2 group. *P* values were calculated using the Student's t‐test (two‐tailed) in (e–g). *P < 0.05, **P <0.01, ***P < 0.001.

Synapses are connections between axons and dendrites of different neurons, which are the basis of information processing and storage in the brain.^[^ ^40^ ^]^ Previous studies have shown that dysfunction of synaptic transmission had a critical role in schizophrenia.^[^ ^41^, ^42^, ^43^, ^44^ ^]^ Of note, morphological alterations of synapses, including the number, density, size, and shape of dendritic spines have been frequently reported in schizophrenia.^[^ ^45^, ^46^, ^47^, ^48^, ^49^ ^]^ It has been reported that the density of spine decreased in schizophrenia due to excessive spine pruning during late childhood or adolescence,^[^ ^49^ ^]^ indicating the pivotal role of synaptic dysfunction in schizophrenia. Dendritic spines can be divided into mushroom, stubby, thin, and branched subtypes based on their shape.^[^ ^50^, ^51^ ^]^ Mushroom spines are regarded as long‐term stable dendritic spines, which mainly correspond to stable and mature synaptic connections. They have larger spine heads, and the size of their spine heads is positively correlated with the size of the excitatory synaptic post‐synaptic density (PSD) and the strength of the synapse.^[^ ^52^, ^53^ ^]^ Thin and stubby are regarded as transient, immature dendritic spines with the property of quick formation and/or elimination.^[^ ^54^, ^55^ ^]^ The dynamic changes of dendritic spines are closely related to learning and memory at the synaptic level. Newly formed dendritic spines (most of them are thin spines) are the structural basis for memory acquisition, and stable dendritic spines (most of them are mushroom spines) are responsible for memory storage. We therefore investigated the role of *Sfmbt1* in spine morphogenesis by knocking‐down *Sfmbt1* expression in primary neurons (**Figure** **8**). We found that *Sfmbt1* knockdown resulted in a significant decrease in the density of stubby, thin, and mushroom spines (Figure 8f). In addition, the proportion of thin spines was also significantly decreased in *Sfmbt1* knocked‐down neurons (Figure 8g). These results recapitulated the synaptic abnormality observed in schizophrenia, indicating that *Sfmbt1* may confer risk of schizophrenia by regulating spine structure and function.

## 3 Discussion

So far, GWASs have identified multiple risk loci that showed robust associations with schizophrenia.^[^ ^7^, ^8^, ^9^, ^10^, ^11^, ^12^, ^13^, ^14^, ^15^ ^]^ Despite the fact that these large‐scale GWASs have provided important insights into the genetic architecture of schizophrenia, how to translate the GWAS findings into biological mechanisms and potential therapeutic targets remain major challenges in the post‐GWAS era. The first step toward mechanistic understanding is to pinpoint the functional (or potential causal) variants from the reported risk loci, a daunting task that is usually impeded by the complex LD pattern and gene regulation. We conducted a functional genomics study on schizophrenia and identified 132 TF binding‐disrupting SNPs in our previous study.^[^ ^22^ ^]^ SNP rs2535629 represents a promising causal variant among the 132 TF binding‐disrupting SNPs. We thus systematically investigated the regulatory mechanism of rs2535629 in this study. We provided convergent and consistent results to prove the functionality of rs2535629 in this study. First, ChIP‐seq and PWM data indicated that rs2535629 is located in a CTCF binding motif with CTCF binding in neuronal cells (Figure 1c,d). Second, FIMO analysis suggested that rs2535629 affected CTCF binding (Figure 1c). Third, we showed that CTCF bound to the sequence containing rs2535629 in vivo and different alleles of rs2535629 altered the binding affinity of CTCF (Figure 2e–g). Fourth, we validated the regulatory effect of rs2535629 using reporter gene assays (Figure 2a–d). These results collectively confirmed that rs2535629 is a functional variant with regulatory effect.

In addition to elucidating the regulatory mechanism of rs2535629, we also identified the potential target genes regulated by rs2535629. Our eQTL analysis showed that rs2535629 was associated with the expression of *GLT8D1*, *NKE4*, and *SFMBT1* in the human brain (Figure 3), suggesting that rs2535629 may confer schizophrenia risk by modulating these genes. We further verified the regulatory effect of CTCF and rs2535629 on these three genes (Figure 4). Of note, we previously showed that *GLT8D1* and *NEK4* might have a role in schizophrenia.^[^ ^29^, ^56^ ^]^ These results indicated that *SFMBT1* might also be one of the potential causal genes (at this risk locus) regulated by rs2535629. We thus characterized the function of *Sfmbt1* in the CNS. We found that *Sfmbt1* not only regulated proliferation and differentiation of NSCs, but also affected the dendritic spine density of neurons, implicating that this gene may be involved in schizophrenia pathophysiology by affecting neurodevelopment and synaptic transmission. It should be noted that we only investigated the effect of *SFMBT1* on the differentiation of NSCs into GFAP positive glia cells and MAP2 positive neurons. Considering there are many cell types (including oligodendrocytes and microglia cells) in the brain, more work is needed to explore if *SFMBT1* affects the differentiation of NSCs into other cell types.

**Figure 9:**
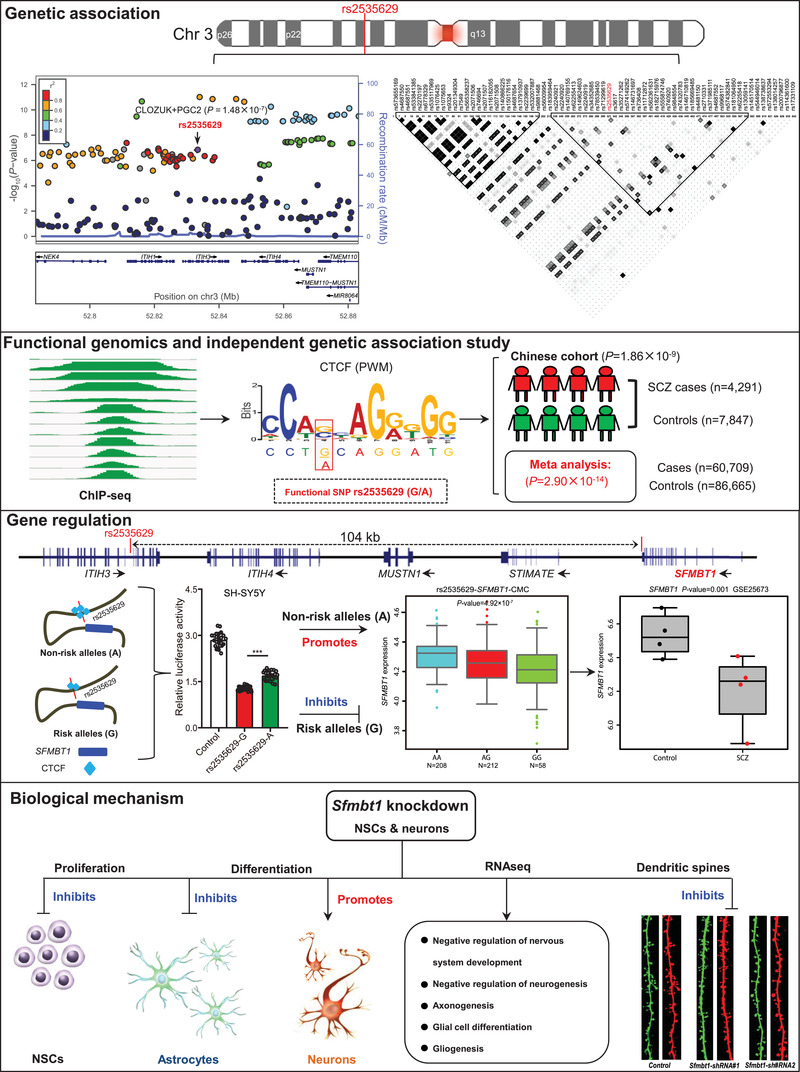
The working model of rs2535629 in schizophrenia pathogenesis. Locuszoom plot revealed the association significances between SNPs located in the 3p21.2 region and schizophrenia. Of note, rs2535629 is located in the seventh intron of *ITIH3* and there are multiple SNPs that showed a high degree of LD with rs2535629. These SNPs had similar association *P* values, making it difficult to pinpoint the causal variant. By using functional genomics, we identified rs2535629 as a regulatory variant in the 3p21.2 risk locus. We validated the regulatory mechanism of rs2535629 with serial experiments, including reporter gene assays, ChIP‐AS‐qPCR, TFs knockdown, and genome editing. In addition, an independent genetic association study further confirmed that rs2535629 was associated with schizophrenia in the Chinese population. rs2535629 physically interacts with *SFMBT1* (by long‐range chromatin interaction) and the schizophrenia risk allele of rs2535629 (G) regulates *SFMBT1* expression by altering CTCF binding, which resulted in lower *SFMBT1* expression. Perturbation of *SFMBT1*expression inhibits proliferation of NSCs and differentiation of NSCs into glial cells, as well as promotes differentiation of NSCs into neurons. *Sfmbt1* expression perturbation also affected genes and pathways associated with neurodevelopmental and synaptic transmission. These results demonstrated that the functional variant rs2535629 in *ITIH3* conferred schizophrenia risk through regulating (via long‐range chromatin interaction) expression of *SFMBT1*, a gene whose expression perturbation affected neurodevelopment.

In addition to *GLT8D1* ^[^ ^29^ ^]^ and *NEK4*,^[^ ^56^ ^]^ our study also suggests that *SFMBT1* may be a potential risk gene at this locus, and functional SNP rs2535629 may confer schizophrenia risk by regulating *SFMBT1*. We noticed that rs2535629 is located in the intron 7 of *ITIH3* gene and the distance between rs2535629 and *SFMBT1* is about 104 kb (**Figure** **9**), suggesting that rs2535629 may regulate *SFMBT1* through long‐range chromatin interaction. We thus examined if rs2535629 interacts with *SFMBT1* in neuronal cells using the 3D‐genome Interaction Viewer and database.^[^ ^57^ ^]^ Intriguingly, rs2535629 physically interacts with *SFMBT1* (but not *NEK4* and *GLT8D1*) in the dorsolateral prefrontal cortex (human) (Figure S6, Supporting Information). More importantly, a recent study by Jung et al. also showed that rs2535629 physically interacts with *SFMBT1* promoter.^[^ ^58^ ^]^ Of note, rs2535629 disrupted the binding of CTCF, a key regulator which mediates chromatin interaction.^[^ ^59^, ^60^ ^]^ These data suggested that rs2535629 regulates *SFMBT1* expression by affecting long‐range chromatin interaction. Consistent with this, our recent transcriptome‐wide association also showed that *SFMBT1* is a schizophrenia risk gene whose expression level change may have a role in schizophrenia.^[^ ^61^ ^]^ These convergent lines of evidence suggest that *SFMBT1* is a potential schizophrenia risk gene at this locus.

*SFMBT1* encodes Scm‐like with four malignant brain tumor repeat (MBT) domains 1, which is postulated to be a histone‐binding protein.^[^ ^62^ ^]^ *SFMBT1* mediates the recruitment of transcriptional inhibition complexes to target genes to repress transcription via compressing chromatin.^[^ ^63^, ^64^ ^]^ *SFMBT1* regulates important cellular functions, including cell differentiation,^[^ ^63^ ^]^ migration^[^ ^65^ ^]^ and gene transcription. Expression analysis showed that *SFMBT1* is widely expressed in human tissues and many brain regions (Figures S7 and S8, Supporting Information).^[^ ^66^, ^67^ ^]^ However, *SFMBT1* expression in brain tissues is relatively low compared with other tissues (Figure S7, Supporting Information). In addition, cell‐type‐specific expression analysis (using the expression data from the UCSC cell browser (https://cells.ucsc.edu/?ds=autism&gene=SFMBT1)) showed that *SFMBT1* expression is relatively high in L2/3 (upper‐layer excitatory neurons), L5/6 (deep‐layer cortico‐cortical excitatory projection neurons), VIP interneurons and parvalbumin interneurons (Figure S9, Supporting Information). Of note, *SFMBT1* copy number abnormality has been identified in patients with brain diseases^[^ ^68^, ^69^ ^]^ and a segmental copy number loss of *SFMBT1* was identified in idiopathic normal pressure hydrocephalus (iNPH).^[^ ^70^ ^]^ The main clinical phenotypes of iNPH patients are gait disorder, cognitive impairment, and urinary incontinence.^[^ ^71^ ^]^ The cerebral structure of iNPH cases also exhibits abnormalities, including enlargement of ventricle, edema of white matter area, and decrease of cerebral blood flow.^[^ ^72^ ^]^ These results suggest that *SFMBT1* may play an important role in the CNS. We noticed that *SFMBT1* knockdown increased neuronal cell differentiation (Figure 6d) but decreased dendritic spine density (Figure 8), suggesting that *Sfmbt1* may have different roles in different neurodevelopmental stages. Of note, the differentiation of neuronal cells and the maturation of synapses occur in different developmental stages of neurodevelopment. The differentiation of neural stem cells into neurons mainly occurs in the prenatal stage, while the maturation of synapses mainly occurs in the early postnatal and adolescent stages.^[^ ^73^, ^74^, ^75^, ^76^, ^77^ ^]^ In addition, we also found that *Sfmbt1* knockdown impaired the proliferation of neural stem cells, and differentiation assays showed that *Sfmbt1* knockdown promoted differentiation of neural stem cells toward neuronal cell fate. Furthermore, *Sfmbt1* knockdown resulted in a significant decrease in dendritic spine density. Considering that *SFMBT1* is a histone‐binding protein that represses transcription by mediating the recruitment of corepressor complexes to target genes, it is likely that this gene may have different roles in the differentiation of neural cells and synaptogenesis (by regulating the expression of target genes).

To explore if *SFMBT1* can be targeted as a potential therapeutic target, we explored the interaction between *SFMBT1* and drugs using the drug‐gene interaction database^[^ ^78^ ^]^ (https://dgidb.genome.wustl.edu/). No interaction between *SFMBT1* and drugs was observed. More work is needed to explore if *SFMBT1* can be served as a potential therapeutic target. Taken together, we provided convergent and consistent lines of evidence that support rs2535629 confers risk of schizophrenia by modulating *SFMBT1* expression, a gene whose expression perturbation might be involved in schizophrenia by affecting neurodevelopment and synaptic morphologies (two key pathogenic characteristics of schizophrenia).

Intriguingly, rs2535629 is located in the intron of *ITIH3*, a gene that was previously reported to be associated with schizophrenia.^[^ ^79^, ^80^ ^]^ In fact, the *ITIH3* locus was one of the earliest schizophrenia risk loci identified by GWASs.^[^ ^79^ ^]^ This risk locus is also one of the best replicated schizophrenia loci in European and Chinese populations.^[^ ^81^ ^]^ Of note, Brandl et al. found that rs2535629 was also significantly associated with antipsychotic response in schizophrenia patients.^[^ ^82^ ^]^ Considering cognitive impairment is a core feature of schizophrenia,^[^ ^83^, ^84^ ^]^ we also explored if rs2535629 was associated with cognition using the genome‐wide associations (N = 269867 individuals) reported by Savage et al.^[^ ^85^ ^]^ Interestingly, rs2535629 also showed a significant association with intelligence (*P* = 2.01 × 10^−7^), with the risk allele (G) associated with poor cognition. These consistent genetic findings strongly suggest this region harbors authentic risk variants for schizophrenia.

In addition to schizophrenia, rs2535629 also showed suggestive association with bipolar disorder (*P* = 4.93 × 10^−7^).^[^ ^24^ ^]^ Considering the high genetic correlation between schizophrenia and bipolar disorder,^[^ ^86^ ^]^ it is possible that rs2535629 may be a common risk variant for schizophrenia and bipolar disorder. rs2535629 might confer risk of bipolar disorder by regulating *SFMBT1*. However, *SFMBT1* expression did not show significant change (*P* = 0.66) in brains of bipolar cases (N = 222) compared with controls (N = 936) (expression data were from the PsychENCODE).^[^ ^27^ ^]^ More work is needed to demonstrate the role of *SFMBT1* in bipolar disorder.

We previously showed that a missense variant (rs3617) in the coding region of *IHTI3* conferred schizophrenia risk by altering the protein abundance and function of *ITIH3*.^[^ ^87^ ^]^ In this study, we identified a novel regulatory variant that might contribute to schizophrenia risk by regulating the expression of the distal gene *SFMBT1*. Our study revealed the complex genetic mechanisms of the *ITIH3* locus in schizophrenia. That is, though both genetic variants in the coding and non‐coding regions of *ITIH3* are associated with schizophrenia, these variants confer risk of schizophrenia via different mechanisms (i.e., risk variants in *ITIH3* coding region confer schizophrenia susceptibility by altering protein function, while risk variants in non‐coding (*ITIH3* intron) regions may contribute to schizophrenia risk by modulating the expression of distal gene (or genes)). Our study also highlights that the gene (or genes) nearest to the genome‐wide significant risk variants are not necessarily the responsible gene for disease susceptibility. It is possible that genetic variants may confer the risk of schizophrenia by regulating distal genes. Thus, the gene (or genes) nearest to the genome‐wide significant risk variants could not be simply designated as the responsible gene.

There are several limitations to this study. First, considering the fact that isolating and culturing human NSCs is much difficult than mNSCs, we used mNSCs to explore the role of *Sfmbt1* in the proliferation and differentiation of NSCs. Using human neural stem cells or neuronal cells (from fetal NSCs or human induced pluripotent stem cells) will facilitate to elucidate the role of *SFMBT1* in human neurodevelopment. In addition, more work (e.g., animal model) is needed to investigate the role and mechanisms of *SFMBT1* dysregulation in schizophrenia pathogenesis. Third, we noticed that the risk allele (G) of rs2535629 is associated with a higher expression level of *NEK4* (Figure 3b) and a lower expression level of *SFMBT1* and *GLT8D1* (Figure 3c,d). The opposite impact of G allele on the expression of *NEK4* compared to *SFMBT1* and *GLT8D1* suggests the complex regulatory mechanism of rs2535629. It is well‐established that gene regulation is a complex process with sophisticated interactions between regulatory elements and TFs. In eQTL analysis, it is usually observed that the reference allele of an SNP is associated with higher expression of some genes and lower expression of other genes.^[^ ^19^, ^26^, ^88^, ^89^, ^90^, ^91^ ^]^ For example, Hua et al. showed that a prostate cancer risk SNP rs11672691 (resides in the promoter of a short isoform of long noncoding RNA *PCAT19* (*PCAT19‐*short) and the third intron of the long isoform (*PCAT19‐*long)) is associated with decreased and up‐regulated expression levels of *PCAT19‐*short and *PCAT19‐*long, respectively.^[^ ^89^ ^]^ Hua et al. showed that this risk SNP mediates promoter‐enhancer switching by affecting the binding of NKX3.1 and YY1, indicating the pleiotropic effects of regulatory elements (i.e., a regulatory element may be acted as an enhancer for some genes, while this regulatory element may also be acted as a repressor for other genes). Besides, interactions between regulatory elements (such as enhancer and promoter) also have a pivotal role in the regulation of gene expression. Moreover, TFs also play a critical role in gene regulation and many studies have revealed the multi‐faceted role of TFs. As a key transcription regulator, CTCF has both transcription activation and repression functions,^[^ ^92^, ^93^, ^94^ ^]^ with a context‐dependent effect. Of note, the interaction between CTCF and other TFs (including YY1^[^ ^95^ ^]^ and RAD21^[^ ^96^ ^]^) also has a crucial role in gene regulation. Last, the effect of DNA fragment deletion (usually several hundred base pairs) may be different from the effect of regulatory variant (single nucleotide). The regulatory effect and mechanism of rs2535629 on *NEK4*, *GLT8D1*, and *SFMBT1* might be different and much complex than imagination. More work is needed to elucidate the mechanism of rs2535629 on *NEK4, SFMBT1*, and *GLT8D1*. Fourth, considering that rs2535629 is associated with the expression of *GLT8D1*, *NEK4*, and *SFMBT1*, we could not rule out the possibility that rs2535629 might confer schizophrenia risk by regulating the expression of *GLT8D1* and *NEK4*. More work is needed to further elucidate the role of rs2535629 and *SFMBT1* in schizophrenia.

In summary, we elucidated the molecular regulatory mechanism of the schizophrenia risk variant rs2535629 at the single‐nucleotide resolution level, and we showed that rs2535629 may confer schizophrenia risk by regulating *SFMBT1* expression (a gene about 104 kb away). Our work demonstrates the complex regulatory mechanism of schizophrenia risk variants (i.e., risk variants in intronic or intergenic regions may exert their biological effects on schizophrenia by regulating distal genes). Our findings not only provide important molecular and biological insights into the genetic regulatory mechanism of schizophrenia, but also provide a framework to identify and elucidate the functional variants from the reported risk loci. Moreover, our study translated the GWAS findings into regulatory mechanism and biology implication, a key process toward mechanisms delineating and development of new drugs and therapeutic approaches.

## 4 Experimental Section

Detailed information about the reagents is provided in Table S1, Supporting Information.

### Schizophrenia Cases and Controls

A total of 4320 schizophrenia cases and 7847 controls were included in the genetic association study. All of the subjects were of Han Chinese ancestry and detailed information about the samples have been described in the previous studies.^[^ ^87^, ^97^, ^98^ ^]^ All of the cases were diagnosed with DSM‐IV criteria, with the use of Structural Clinical Interview for DSM (SCID) mental disorders. Detailed information about clinical interviews is provided in Supporting Information Material. Healthy controls were recruited from the general population and information about their medical and family psychiatric histories were screened. Informed consent were obtained from all of the participants and the research protocol was approved by the Internal Review Board of the Kunming Institute of Zoology, Chinese Academy of Sciences. Genomic DNA was extracted from peripheral blood using phenol‐chloroform method.

### Genotyping

Genotyping was performed using the SNaPshot method and detailed procedures have been described in the previous studies.^[^ ^87^, ^98^ ^]^ Primer sequences for PCR and genotyping are provided in Tables S2 and S3, Supporting Information.

### Association and Meta‐Analysis

The association between rs2535629 and schizophrenia was performed with PLINK.^[^ ^99^ ^]^ The meta‐analysis was conducted by using PLINK.

### Cell Culture

HEK‐293T (a tumor cell line from human embryonic kidneys), SK‐N‐SH (a human neuroblastoma cell line), SH‐SY5Y (thrice cloned subline of the SK‐N‐SH), and U251 (a human glioma cell line) (all originally from ATCC) were purchased from the cell bank of Shanghai Institute of Cell and Biochemistry, Chinese Academy of Sciences. These cells were cultured as previously described.^[^ ^87^, ^100^, ^101^ ^]^ PCR was used for mycoplasma detection and primer sequences are provided in Table S4, Supporting Information. Detailed information about cell culture is provided in Supporting Information Material.

### Dual Luciferase Reporter Gene Assays

To investigate the regulatory effect of rs2535629, dual‐luciferase reporter gene assays were conducted. The sequences containing different alleles of rs2535629 were cloned into pGL‐3 promoter vector, which was used to test regulatory activity (enhancer or repressor) of the inserted element (Table S5, Supporting Information). Detailed information about reporter gene assays is provided in Supporting Information material.

### Chromatin Immunoprecipitation (ChIP)‐qPCR

ChIP‐qPCR was performed using the SimpleChIP Enzymatic Chromatin IP Kit from Cell Signaling, following the manufacturer's instructions. Briefly, cells were cross‐linked with 1% formaldehyde for 10 min at room temperature and quenched with the 125 mm glycine, then centrifuged for 5 min (2000 g) at 4 °C. After aspirating the supernatant, pellets were collected and re‐suspended in precooled buffer A (containing DTT and PIC). After incubating for 10 min on ice (inverted every 3 min), cells were centrifuged at 2000 g for 5 min to isolate nuclei. The isolated nuclei were then washed twice with cold buffer B (containing DTT), and an appropriate amount of micrococcus nuclease was added to digest the DNA into 150–900 bp fragments (20 min at RT). EDTA was used to stop the reaction. After centrifuging at 16 000 g for 1 min and aspirating the supernatant, pellets were re‐suspended in 1 × ChIP buffer (containing PIC) and incubated for 10 min on ice. Sonication was performed to break the nuclear membrane (3 mm probe, 60 W ultrasonic power, 20 s each time, 3 times in total) followed with centrifugation at 9400 g for 10 min at 4 °C (to remove the nuclear fragments). The supernatant contains cross‐linked chromatin fragment. A total of 50 µL chromatin samples were purified and agarose gel electrophoresis was used to detect the efficiency of enzyme digestion (Figure S10a, Supporting Information). The concentration of DNA was measured for subsequent experiments.

Cross‐linked chromatins (8 µg chromatin DNA per reaction) were used for the experimental group (CTCF antibody) and IgG was used for negative control group. The corresponding antibody for target protein was added into each sample and served as experimental group, and IgG was added as negative control. After incubating at 4 °C (on the rotor) for overnight, the enriched chromatins were collected using ChIP‐grade protein G magnetic beads. DNA was purified for subsequent qPCR analysis.

According to the DNA sequence of rs2535629, primers for ChIP‐qPCR and ChIP‐AS‐qPCR were designed (Table S6, Supporting Information). Primers for ChIP‐qPCR were designed to be located within the up and downstream 100 bp from rs2535629. The terminal base of one of the primers for ChIP‐AS‐qPCR was located at rs2535629, and the penultimate base was replaced with an unpaired base to ensure the specificity of the primer. The length of the amplified fragment was appropriate within 100 bp (Figure S10b, Supporting Information). The qPCR system and enrichment efficiency were calculated according to the instructions of the SimpleChIP Enzymatic Chromatin IP Kit from Cell Signaling (#9003):

20 µL reaction system: 2 µL ChIP‐DNA/input DNA; 2 µL primer (5 µM); 10 µL qPCR Master Mix; 6 µL Water.

(1) $$\mathrm{Enrichment}\;\mathrm{percentage}=2\%\times 2^{\left(C[T]\;\mathrm{Input}\;\mathrm{Sample}-C[T]\;\mathrm{IP}\;\mathrm{Sample}\right)}$$

(2) $$C\left[T\right]=C_{T}=\mathrm{Threshold}\;\mathrm{cycle}\;\mathrm{of}\;\mathrm{PCR}\;\mathrm{reaction}$$

The enrichment efficiency of rs2535629 for CTCF was obtained by qPCR, and the results were verified by Sanger sequencing.

### CRISPR‐Cas9‐Mediated Genome Editing

The online sgRNA design tool (https://zlab.bio/guide‐design‐res) was used to design the sgRNAs (with the use of default settings and parameters) and sgRNAs with high scores were used (Table S7, Supporting Information). Detailed information about genome editing is provided in Supporting Information Material.

### Knockdown Assays

shRNAs targeting the studied genes were designed with the online design tool (https://rnaidesigner.thermofisher) and shRNAs with the highest scores were used. (Table S8, Supporting Information). The primers for knockdown efficiency test are provided in Table S9, Supporting Information. Detailed information about knockdown assays is provided in Supporting Information Material.

### Isolation and Culturing of Mouse NSCs

Mouse NSCs were isolated and cultured as previously described.^[^ ^17^, ^29^, ^87^ ^]^ Briefly, brains of embryonic mice (E13.5) were dissected and pipetted repeatedly to dissociate the tissues. The dissociated cells were cultured in DMEM/F12 medium containing N2, B27, EGF, bFGF, and heparin.

### Proliferation Assays

Proliferation assays were conducted as previously described.^[^ ^17^, ^19^, ^87^ ^]^ In brief, 4 × 10^5^ cells were plated into each well of the 12‐well plates. After culturing for 48 h (when the confluence reached about 70–80%), BrdU was added to the medium at a final concentration of 10 µg/mL. After incubating for 30 mins, the cells were washed with PBS and fixed with 4% paraformaldehyde (PFA) at room temperature for 15 min. The fixed cells were permeabilized with 0.3% PBST and blocked with 5% bovine serum albumin (BSA) for 1 h at room temperature. Immunofluorescence assays were conducted to detect and quantify the BrdU incorporated cells as previously described.^[^ ^17^, ^19^, ^87^, ^100^ ^]^ In addition to BrdU incorporation assays, CCK8 assay was also carried out to determine the proliferation ability of the cells (as described in the previous studies).^[^ ^17^, ^19^, ^87^, ^100^ ^]^

### Differentiation of NSCs

Differentiation assays were carried out as previously described.^[^ ^19^, ^87^ ^]^ In brief, 4 × 10^5^ cells/per well were seeded into 12‐well plates and cultured in a proliferation medium overnight. The cells were washed with PBS and the proliferation medium was replaced with a differentiation medium: DMEM medium supplemented with 1 × B27 (without vitamin A) and 10% FBS. After spontaneous differentiation for three days, cells were rinsed with PBS once and fixed with 4% PFA for subsequent immunofluorescence experiments.

### Assays of Dendritic Spine Density

Rat primary neurons were isolated from fetal brains (embryonic day 17.5 to 18.5). Briefly, brain tissues were dissected and washed in a pre‐cooled separation buffer (HBSS + 1% HEPES, with 50 units/mL of penicillin and 50 µg/mL of streptomycin). The tissue blocks were digested with digestion buffer (Neurobasal + 2% B27 + 1% Glutamax + 2 mg/mL Papain + 5U mL^−1^ DNase I) for 18 min (at 37 °C). During the digestion process, tissues were gently shaken every 3–5 min (to make the tissue blocks in full contact with the digestion dilution and avoid the adhesion caused by DNA). Neuron Chow solution (Neurobasal + 2% B27 + 1% Glutamax) (with 2.5% heat‐inactivated Fetal bovine serum (FBS)) was used to terminate digestion and to re‐suspend digested tissue. A sterilized pipette was used to dissociate the digested tissue (by repeated pipetting). The dissociated cells were filtered to obtain a single cell. The isolated neurons were seeded into the PDL (Poly‐D‐lysine hydrobromide) (50 µg/mL PDL) pre‐coated dishes at a density of 4 × 10^5^ /well (6‐well plates) and cultured in Neuron Chow medium. After culturing for 14 days, the recombinant plasmid (pSicoR‐Ef1a‐mCh‐Puro‐shRNA) (3 µg) and the Venus (1 µg) were co‐transfected into the cultured neurons using Lipofectamine 3000. Three days post‐transfection, cells were fixed and immunofluorescence staining was conducted to evaluate the impact of *Sfmbt1* knockdown on dendritic spines. Detailed information about immunofluorescence staining (including antibodies, image capture, and processing) are provided in the Supporting Information material. More than 20 neurons in each group were analyzed for morphological analysis of dendritic spines (including thin, mushroom, and stubby spines).^[^ ^51^, ^102^ ^]^ Image J software^[^ ^103^ ^]^ (https://imagej.nih.gov/ij/) and NeuronStudio^[^ ^104^ ^]^ (https://m.vk.com/neuron_studio) were jointly used for morphological analysis. The detailed processes for classifying different types of spines are provided in Supporting Information materials. The statistical differences between *Sfmbt1* knockdown groups and the control group were determined by a two‐tailed Student's t‐test, with the significance threshold was set at *P*<0.05.

### Statistical Analysis

Statistical analyses were performed using the GraphPad Prism 8.0 software. *P* values were calculated using the Student's t‐test (two‐tailed). *P* < 0.05 was considered as significant. Quantitative data were expressed as mean ± standard deviation (S.D.). The exact values of sample size (n) and the name of each statistical test are provided in each figure legend. No statistical tests were conducted to predetermine sample sizes.

## Conflict of Interest

The authors declare no conflict of interest.

## Author Contributions

Y.L., C.M., and S.L. contributed equally to this work. X.J.L. conceived, designed, and supervised the whole study. Y.F.L., C.G.M., J.Y.W., K.Q.L., J.L., D.H., R.C., S.W.L., and Y.X.L. extracted the genomic DNA. Y.F.L. performed genotyping assays, reporter gene assays, ChIP‐qPCR, knockdown assays, and genome editing. X.Y.L., J.W.L., and J.F.Y. performed the data analysis. Y.F.L. and C.G.M. performed proliferation and differentiation of mNSCs assays. Y.F.L. and S.W.L. isolated the primary rat neurons, cultured the neurons, and conducted the morphological analyses (dendritic spine density of neurons). L.Y.F. and C.G.M. wrote the draft of the manuscript. X.J.L. oversaw the project and finalized the manuscript. W.Q.L., Y.F.Y., and L.X.L. contributed to this work in data generation, analysis, and the interpretation of the results. X.X. and M.L. provided advice and discussion during the design and practice of the current study. All authors revised the manuscript critically and approved the final version.

## Supporting information

Supporting Information

## Data Availability

The data that support the findings of this study are available from the corresponding author upon reasonable request.
