# Supplementary material for: Regulatory Variant rs2535629 in ITIH3 Intron Confers Schizophrenia Risk By Regulating CTCF Binding and SFMBT1 Expression
Source: Adv Sci (Weinh). 2022 Jan 2;9(6):2104786. doi: 10.1002/advs.202104786 (PMC8867204; doi:10.1002/advs.202104786)
Supplement: Supplementary file 1 — Supporting Information [file ADVS-9-2104786-s001.pdf]

ADVANCED SCIENCE
Open Access

Supporting Information

for *Adv. Sci.*, DOI: 10.1002/advs.202104786

Regulatory variant rs2535629 in *ITIH3* intron confers schizophrenia risk by regulating CTCF binding and *SFMBT1* expression

Yifan Li, Changguo Ma, Shiwu Li, Junyang Wang, Wenqiang Li, Yongfeng Yang, Xiaoyan Li, Jiewei Liu, Jinfeng Yang, Yixing Liu, Kaiqin Li, Jiao Li, Di Huang, Rui Chen, Luxian Lv, Xiao Xiao, Ming Li, and Xiong-Jian Luo[*]

Supplementary material for

Regulatory variant rs2535629 in *ITIH3* intron confers schizophrenia risk by regulating CTCF binding and *SFMBT1* expression

Yifan Li[1,2,9], Changguo Ma[1,6,9], Shiwu Li[1,2,9], Junyang Wang[1,2], Wenqiang Li[3,4], Yongfeng Yang[3,4], Xiaoyan Li[1,5], Jiewei Liu[1], Jinfeng Yang[1,2], Yixing Liu[1,2], Kaiqin Li[1,2], Jiao Li[1,2], Di Huang[1], Rui Chen[1,2], Luxian Lv[3,4], Xiao Xiao[1], Ming Li[1], Xiong-Jian Luo[1,2,7,8,*]

[1]Key Laboratory of Animal Models and Human Disease Mechanisms of the Chinese Academy of Sciences & Yunnan Province, Kunming Institute of Zoology, Chinese Academy of Sciences, Kunming, Yunnan 650204, China

[2]Kunming College of Life Science, University of Chinese Academy of Sciences, Kunming, Yunnan 650204, China

[3]Henan Mental Hospital, The Second Affiliated Hospital of Xinxiang Medical University, Xinxiang, Henan 453002, China;

[4]Henan Key Lab of Biological Psychiatry, International Joint Research Laboratory for Psychiatry and Neuroscience of Henan, Xinxiang Medical University, Xinxiang, Henan 453002, China

[5]Key Laboratory of Intelligent Computing and Signal Processing of Ministry of Education, Institutes of Physical Science and Information Technology, Anhui University, Hefei, Anhui 230601, China

[6]Yunnan Key Laboratory for Basic Research on Bone and Joint Diseases & Yunnan Stem Cell

Translational Research Center, Kunming University, Kunming, Yunnan 650214, China

[7]Center for Excellence in Animal Evolution and Genetics, Chinese Academy of Sciences, Kunming, Yunnan 650204, China

[8]KIZ-CUHK Joint Laboratory of Bioresources and Molecular Research in Common Diseases, Kunming Institute of Zoology, Chinese Academy of Sciences, Kunming, Yunnan 650204, China

[9]These authors contributed equally to this work

*To whom correspondence should be addressed: Xiong-Jian Luo, Key Laboratory of Animal Models and Human Disease Mechanisms, Kunming Institute of Zoology, Chinese Academy of Sciences, Kunming, Yunnan 650204, China; Tel: +86-871-68125413, Fax: +86-871-68125413, E-mail: luoxiongjian@mail.kiz.ac.cn (XJL).

Detailed methods are provided in this paper and include the following:

- **METHOD DETAILS**

  Genotyping

  Cell culture

  Schizophrenia cases and controls

  Dual luciferase reporter gene assay

  CRISPR-Cas9-mediated genome editing

  Knockdown assays

Assays of dendritic spine density

Detailed information about the reagents are provided in the **Supplementary Table 1.**

Supplementary Methods

Genotyping

In brief, the genomic sequence surrounding the rs2535629 was amplified by PCR with specific primers (**Supplementary Table 2**). The PCR products were then treated with shrimp alkaline phosphatase (SAP) and exoenzyme I (ExoI) to remove unincorporated dNTPs and primers. The purified products were used as template to genotype rs2535629 with snapshot multiple mixing solution, genotyping primers (**Supplementary Table 3**) and ddNTPs. Genotyping data were analyzed and manually checked using the GeneMapper software 4.0.

Cell culture

HEK-293T and U251 cells were cultured in high-glucose Dulbecco's Modified Eagle's medium containing 10% FBS, 1× penicillin and streptomycin (contains 50 units/mL of penicillin and 50 μg/mL of streptomycin). SH-SY5Y and SK-N-SH was cultured in high-glucose DMEM supplemented with 10% FBS, 10 mM sodium pyruvate solution, 1× Minimum Essential Medium non-essential amino acid solution, penicillin and streptomycin. Cells were cultured at 37℃ (with 5% CO_2) and there were no mycoplasma contamination detected in our study (**Supplementary Table 4)**.

Schizophrenia cases and controls

All of cases were diagnosed with DSM-IV criteria, using the Structured Clinical Interview for DSM-IV (SCID)[1]. The detailed information about the onset of schizophrenia, the duration course, symptoms, family history of psychiatric illnesses and medical history were evaluated by at least two independent experienced psychiatrists to reach a consensus DSM-IV diagnosis. Subjects with a history of alcoholism, epilepsy, neurological disorders, or drug abuse were excluded from the study.

Dual luciferase reporter gene assays

The human DNA sequence containing rs2535629 was amplified with specific primers (**Supplementary Table 5**). The amplified products were digested with restriction enzymes KpnI and XhoI, then ligated into the pGL3-promoter vector. The ligated vectors were transfected into DH-5α cells and selected with LB culture plates containing ampicillin. After screening for 18 hours, single colonies were selected for further amplification culture. The sequence of the inserted DNA was verified by Sanger sequencing. PCR-mediated point mutation technology was used to generate DNA fragments containing alternative alleles of SNP **(Supplementary Table 5)**.

Luciferase reporter gene assays were performed in HEK-293T, SH-SY5Y, SKN-SH and U251 cells. For HEK-293T cells, 4×10^4 cells were seeded into 96-well plates. After culturing for 24 hours, the recombinant plasmid (100 ng) and the internal control plasmid pRL-TK (Renilla Luciferase) (20 ng) were co-transfected into the cells using Lipofectamine 3000. For SK-N-SH and SH-SY5Y, and U251 cells, 6×10^4 cells were plated into a 96-well plate. After 24 hours culture, the recombinant plasmid (150 ng) and the internal control plasmid pRL-TK (50 ng) were co-transfected into the cells using Lipofectamine 3000. 48 hours post transfection, luciferase activity was determined by using Luminoskan Ascent instrument (Thermo Scientific) and the dual-luciferase reporter gene detection system. The testing process is carried out in accordance with the manufacturer's recommended instructions.

CRISPR-Cas9-mediated genome editing

To knockout the genomic sequence containing rs2535629, we designed two sgRNAs (located in upstream and downstream of rs2535629, respectively) and cloned the sgRNAs into PX-459 vector. SH-SY5Y cells were seeded into a 6-well plate with a density of 1×10^6/well. After culturing for 24 hours, these cells were transfected with the constructed plasmids using Lipo3000. Puromycin (1 μg/ml) was used to select the transfected cells. Genomic DNA was extracted by phenol/chloroform method and the extracted DNA was used as the template for knockout efficiency validation. RNA was extracted by TRIZOL reagent. Takara reverse transcription kit was used for reverse transcription, according to the manufacturer’s instructions. Gene expression was quantified using SYBR kit.

Knockdown assays

The synthesized shRNAs were annealed and ligated into the pLKO.1-EGFP-Puro vector. The lentiviruses were packed with HEK-293T cells cultured in 10 cm culture dish. The constructed shRNA plasmids and the package plasmids were co-transfected into HEK-293T cells when confluence reached about 70%. After culturing for 48 hours, lentiviruses were collected and used to infect the cells. 48 hours post infection, 2 μg puromycin was added to each well for 7 days to kill the uninfected cells. TRIZOL was used to extract total RNA and reverse transcription was conducted using the Takara reverse transcription kit. Gene expression was quantified using the SYBR kit.

Assays of dendritic spine density

Two softwares (ImageJ software[2] (https://imagej.nih.gov/ij/), NeuronStudio[3] (https://m.vk.com/neuron_studio)) were jointly used for neuron morphological analysis. Briefly, the neurons that co-labeled with green (Venus protein which used to manifest spine morphology of neurons) and red (mCherry indicated that the knock-down vectors were transfected into the target neuron) fluorescence were selected for morphological analysis with double-blind. Image J software was used to transform the format of target picture (.czi) produced by Laser confocal instrument (ZEISS) into other format (.tiff) with 8-bit (which can be opened with NeuronStudio software). The NeuronStudio (a toolkit for neurite spine analysis) was then used to trace the second and tertiary dendrite ((total length of 100 μm per neuron)) of target neurons and identify spine types automatically. The derived spine types were confirmed by manual correction according to the 3D structure of neurons and the specific criterion of spines (mushroom: head to neck diameter ratio>1.5, stubby: head to neck diameter ratio＜1.5 and length to neck diameter ratio＜2, thin: head to neck diameter ratio＜1.5 and length to neck diameter ratio>2)[4, 5].

Supplementary Table 1

REAGENT or RESOURCE	SOURCE	IDENTIFIER
**Antibody**		

Anti-MAP2	Merck millipore	Cat# AB5622
Anti-GFAP	Abcam	Cat# ab10062
CTCF (D31H2) XP® Rabbit mAb	Cell Signaling Technology	Cat# 3418
Donkey anti-rabbit 488	Invitrogen	Cat# A32790
Donkey anti-mouse 555	Invitrogen	Cat# A32773
Donkey anti-goat 647	Invitrogen	Cat# A32849
Donkey Anti-Chicken	Jackson	Cat# 703-225-155
Anti-SOX2	Santa cruz	Cat# sc-17320
Anti-NESTIN	Merck	Cat# MAB353
Anti-PAX6	Merck	Cat# ab2237
Chicken polyclonal GFP antibody	Abcam	Cat# ab13970
Rabbit anti-mCherry antibody	Gene Tex	Cat# GTX128508
Bromodeoxyuridine	Novus	Cat# NE500-169
**Bacterial and Virus Strains**		
DH5α Chemically Competent cells	Tsingke	Cat# TSV-A07
Stbl3 Chemically Competent cells	Tsingke	Cat# TSC06
**Chemicals, Peptides, and Recombinant Proteins**		
High-glucose DMEM	Gibco	Cat# C11995500BT
High-glucose DMEM	Gibco	Cat# C12430500BT
DMEM/F-12 medium(mouse NSCs)	Gibco	Cat# 11320033
Fetal Bovine Serum(FBS)	Gibco	Cat# 10091148
Penicillin/Streptavidin	Gibco	Cat# 15070063
Non-essential amino acids	Gibco	Cat# 11140050
Sodium pyruvate solution	Gibco	Cat# 11360070
B-27™ Supplement (50X), minus	Gibco	Cat# 12587010
B-27™ Supplement (50X), serum free	Gibco	Cat# 17504044
N2	Gibco	Cat# 17502048
Neurobasal® Medium	Gibco	Cat# 21103049
Glutmax	Gibco	Cat# 35050061
Laminin	Sigma	Cat# L2020
Poly-D-lysine hydrobromide	SIGMA	Cat# P6407
HBSS	ThermoFisher	Cat# 14175079
HEPES	Solarbio	Cat# H8090
Epidermal Growth Factor(EGF)	STEMCELL	Cat# 78006
Basic Fibroblast growth factor(bFGF)	STEMCELL	Cat# 78003
heparin	STEMCELL	Cat# 7980
SoSoo Mix	TSINGKE	Cat# TSV-S1

Lipofectamine 3000	Thermo Fisher Scientific	Cat# L3000015
Lenti-X Concentrator	Takara	Cat# 631232
TRIzol™ LS Reagent	ThermoFisher	Cat# 15596018
TB Green™ Premix Ex Taq™ II	Takara	Cat# RR820A
XhoI	FastDigest	Cat# FD0694
KpnI	FastDigest	Cat# FD0524
DAPI	Beyotime	Cat# C1002
SimpleChIP® qPCR mix	Cell Signaling Technology	Cat# 88989
**Critical Commercial Assays**		
PrimeScript™ RT reagent Kit with gDNA	Takara	Cat# RR047A
Enhanced Cell Counting Kit-8	Beyotime	Cat# C0042
SimpleChIP® Enzymatic Chromatin IP	Cell Signaling Technology	Cat# 9003S
Dual-Luciferase® Reporter Assay System	Promega	Cat# E1980
**Experimental Models: Cell Lines**		
HEK-293T	ATCC	Cat# CRL-11268
SH-SY5Y	ATCC	Cat# CRL-2266
SK-N-SH	ATCC	Cat# HTB-11
U251	ATCC	Cat# 300385/p534
mNSCs	This paper	N/A
neuron	This paper	N/A
**vectors**		
pLKO.1-EGFP-Puro vector	qincheng BIO	Cat# QCP1717
pSicoR-Ef1a-mCh-Puro	Addgene	Cat# 31845
PX-459	Feng Zhang Lab at MIT	N/A
pRL-TK	Promega	Cat# E2241
pGL3-promoter	Promega	Cat# E1761
pMD2.G	Addgene	Cat# 12259
pSPAX2	Addgene	Cat# 12260
**Software**		
The 3D-genome Interaction Viewer and	Yang et al., 2018	https://www.kobic.kr/3div/
CRISPR sgRNA design tool	Feng Zhang Lab at MIT	https://zlab.bio/guide-design-resource
Knockdown assay-shRNAs design tool	ThermoFisher	https://rnaidesigner.thermofisher.com/
PLINK (v1.09)	Purcell et al., 2007	N/A
GeneMapper software 4.0	ThermoFisher	N/A
ImageJ	NIH	https://imagej.nih.gov/ij/
NeuronStudio	Rodriguez et al., 2006	https://m.vk.com/neuron_studio

Supplementary Table 2. PCR primers used to amplify the genomic sequence containing the rs2535629 (for genotyping assay)

Primer(5’>3’)	rs2535629
rs2535629-F(287bp)	TAGACCAACAGCGTTCATGC
rs2535629-R(287bp)	GAAAAAGACGTTCCTATCCCA

Supplementary Table 3. SNaPSHOT primer used to genotype rs2535629

Primer (5’>3’)	rs2535629
Ex-rs2535629	TTTTTTTTTTTTTTTTTTTTTTTTTTTTTTTTTTTTTTTTTTTTTCAGCCCAAGGCTGTTTTCCT

Supplementary Table 4. PCR primers for detection of mycoplasma contamination

PCR primer (5’>3’)	Mycoplasma primer
F (Forward)	GCATTGGAAACTGTTAGTCTGGAG
R (Reverse)	ATTCCGTTTGAGTTTCATTCTTGC

Supplementary Table 5. PCR primers used for amplification of DNA sequence containing rs2535629 (for reporter gene assay)

PCR primer (5’>3’)	rs2535629
rs2535629-F	CCAGAACATTTCTCTATCGATAGGTACCGCCCAGCTTAGACCAACAGCG
rs2535629-R	GATGCAGATCGCAGATCTCGAGGCCACGTTCTTAGGCACCACT
rs2535629-F-Mut-G>A	CCTACAGGATGTGGGACAGCTG
rs2535629-R-Mut-G>A	CTGTAGGAAAACAGCCTTGGGC

Supplementary Table 6. ChIP-qPCR primer used for CTCF enrichment analysis

ChIP-qPCR primer (5’>3’)	rs2535629
rs2535629-enrichment-F	CTGGATCTAGTGCCATCACCC
rs2535629-enrichment-R	GCTCTAGGCCATCAGCTGTC
rs2535629-AS-qPCR-F-G	GCCCAAGGCTGTTTTCCAG

rs2535629-AS-qPCR-F-A	GCCCAAGGCTGTTTTCCAA
rs2535629-AS-qPCR-R	AGAAGCCAGACATTTACCAG

Supplementary Table 7. sgRNA primers used to knockout DNA fragments containing rs2535629

CRISPR-Cas9-knockout-sgRNA (5’>3’)	
rs2535629-sgRNA-up-F	ACATGAACGAGGCCAAGCGG
rs2535629-sgRNA-up-R	CCGCTTGGCCTCGTTCATGTc
rs2535629-sgRNA-down-F	ACGGACACTACCAAGGAGC
rs2535629-sgRNA-down-R	GCTCCTTGGTAGTGTCCGTc
rs2535629-knockout-F	AAGCCCAGCTTAGACCAACAG
rs2535629-knockout-R	TGTGCCACTGGTGCTGATTA

Supplementary Table 8. Sequences of shRNAs used to knockdown CTCF and *Sfmbt1*

Knockdown-shRNA-primer (5’>3’)	
CTCF-human-shRNA-1	GCAAGACATGCTGATAATTGT
CTCF-human-shRNA-2	GCGAAAGCAGCATTCCTATAT
Sfmbt1-mouse-shRNA-1	GACCATACAAGGCGATCAT
Sfmbt1-mouse-shRNA-2	GCAAGTTATTGGAACCCAT
Sfmbt1-rat-shRNA-1	GCTGCAGTATGAAGGACTT
Sfmbt1-rat-shRNA-2	GCAGTGGAGTCTAAAGAAT

Supplementary Table 9. PCR primers for qPCR

qPCR-primer (5’>3’)	
ACTIN-F	CATGTACGTTGCTATCCAGGC
ACTIN-R	CTCCTTAATGTCACGCACGAT
GAPDH-F	TTCCTACCCCCAATGTATCCG
GAPDH-R	CATGAGGTCCACCACCCTGTT
CTCF-human-qPCR-F:	TCAGTGCAGTTTGTGCAGTTA
CTCF-human-qPCR-R:	TTCCCCTGAATGGGTTCTCAT
GLT8D1-human-qPCR-F:	AAATGCTCTCCGACATGCAGT
GLT8D1-human-qPCR-R:	TTGGAGCGAGTGTTGTGCTG
NEK4-human-qPCR-F：	ACATCATCAAAGTAGGGGACCT

NEK4-human-qPCR-R：	CAGGGCTCATGTAGTAGGGTG
SFMBT1-human-qPCR-F：	GGCTCTGGTATGGAAGAGGTA
SFMBT1-human-qPCR-R：	TCTCAGGATCTGTTCTCACAGC
Sfmbt1-mouse-qPCR-F：	TCCACCACAGTTCCCTACG
Sfmbt1-mouse-qPCR-R：	AACCCAATAAGTCTCAGGGTCA
Sfmbt1-rat-qPCR-F：	GGTCTCCTTTTGGGATTTCT
Sfmbt1-rat-qPCR-R：	GAGGAGGGTTGATGTGTAAA
Map2-mouse-qPCR -F	GCCAGCCTCGGAACAAACA
Map2-mouse-qPCR -R	GCTCAGCGAATGAGGAAGGA
Gfap-mouse-qPCR -F	CGGAGACGCATCACCTCTG
Gfap-mouse-qPCR -R	AGGGAGTGGAGGAGTCATTCG
Tuj1-mouse-qPCR F	TAGACCCCAGCGGCAACTAT
Tuj1-mouse-qPCR -R	GTTCCAGGTTCCAAGTCCACC

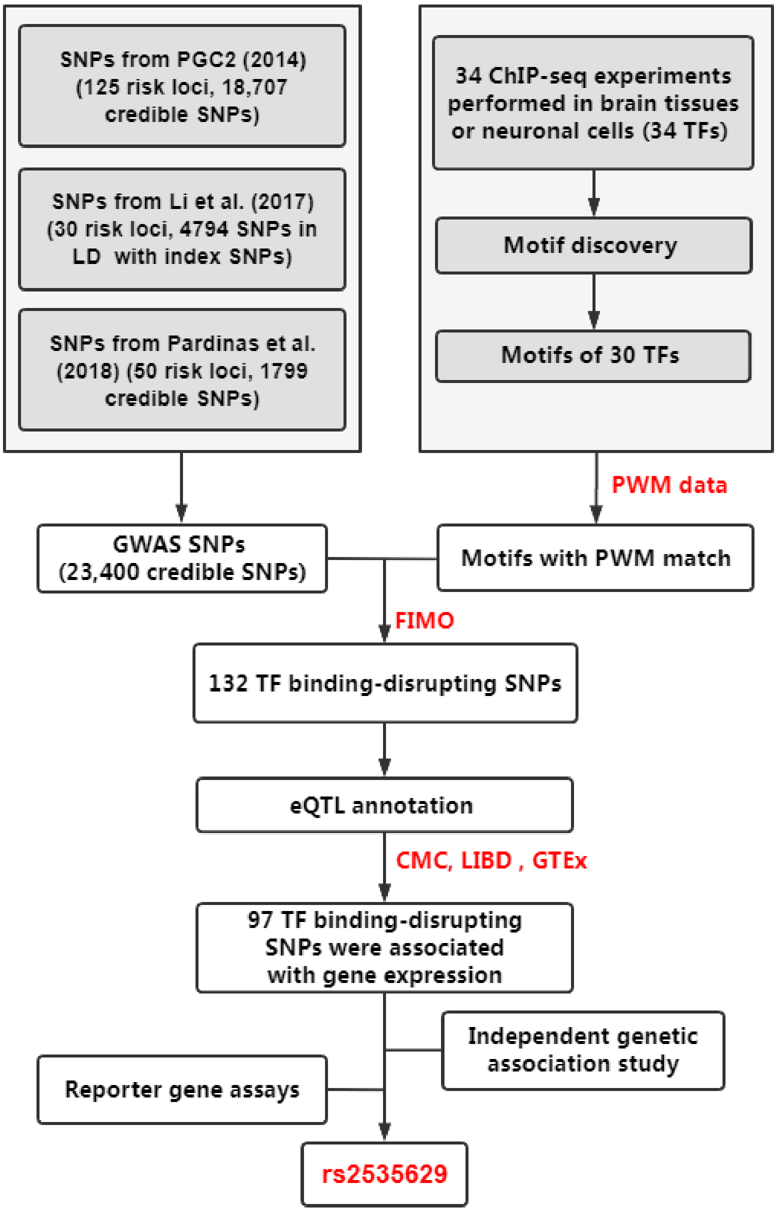

Supplementary Figure 1. Prioritization and identification of regulatory SNPs at schizophrenia risk loci. Risk SNPs from three large-scale GWASs [6-8] were subjected to functional genomics analyses. Chromatin immunoprecipitation and sequencing (ChIP-Seq) experiments performed in brain tissues or neuronal cell lines (such as neuroblastoma) were used to derive the binding motifs (i.e., specific DNA sequence) of corresponding transcription factors. The identified PWMs were then compared to the PWM database, and the matched PWMs (i.e., PWMs derived from ChIP-Seq were compared with the corresponding PWMS in PWM database, and the one with best motif match was used for further analysis) were used to investigate if the risk SNPs were located in the identified PWMs and if different alleles of the SNPs disrupted (or affected) the binding of transcription factor. Expression quantitative trait loci (eQTL) annotation was performed to explore the potential target genes of the identified regulatory SNPs. Reporter gene assays were used to validate the regulatory effects of the identified TF binding–disrupting SNPs, and independent genetic association study was conducted to validate if the identified TF binding-disrupting SNPs were associated with schizophrenia in Chinese population.

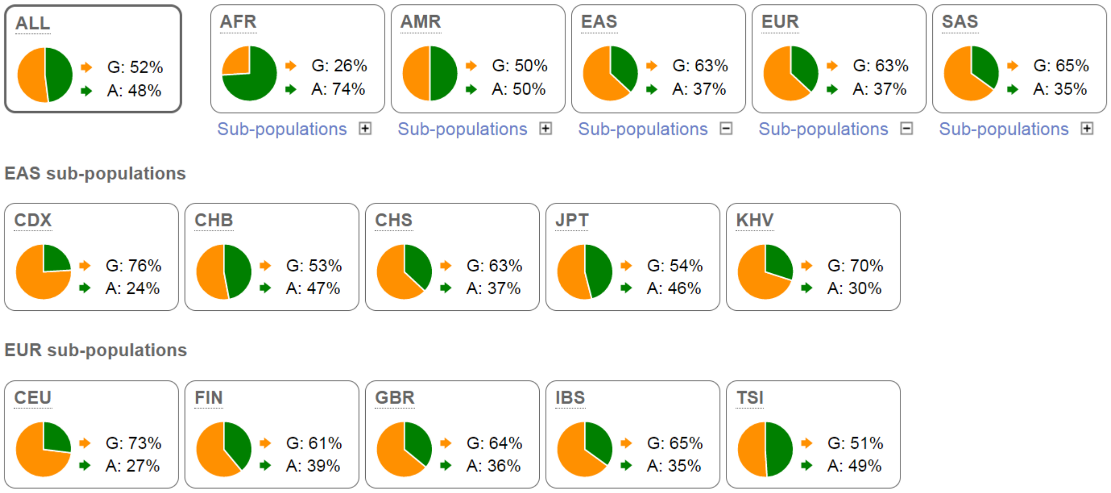

Supplementary Figure 2. Allelic frequency of rs2535629 in differently populations. Upper panel: AFR: African, AMR: American, EAS: East Asian, EUR: South Asian. Middle panel: CDX: Chinese Dai in Xishuangbanna, CHB: Han Chinese in Beijing, CHS: Southern Han Chinese, JPT: Japanese in Tokyo, KHV: Kinh in Ho Chi Minh City. Bottom panel: CEU: Utah residents with Northern and Western European ancestry, FIN: Finnish in Finland, GBR: British in England and Scotland, IBS: Iberian populations in Spain, TSI: Toscani in Italy. Data were from the Ensembl website (http://asia.ensembl.org/Homo_sapiens/Variation/Population?db=core;r=3:52798703-52799703;v=rs2535629;vdb=variation;vf=91992884#population_freq_EUR).

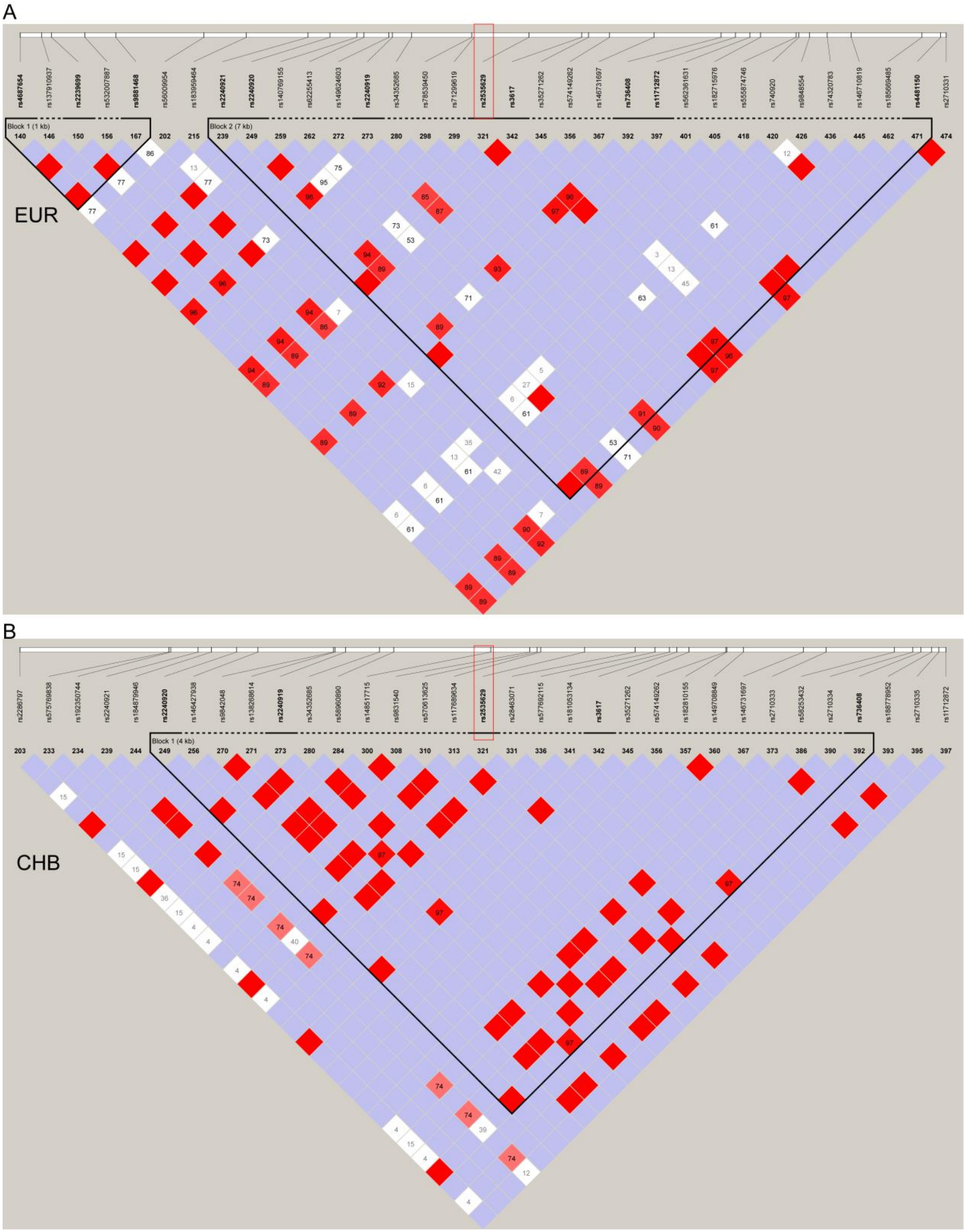

Supplementary Figure 3. LD pattern of the genomic region surrounding rs2535629 in Europeans and East Asians. (genotype data were from the 1000 Genomes project).

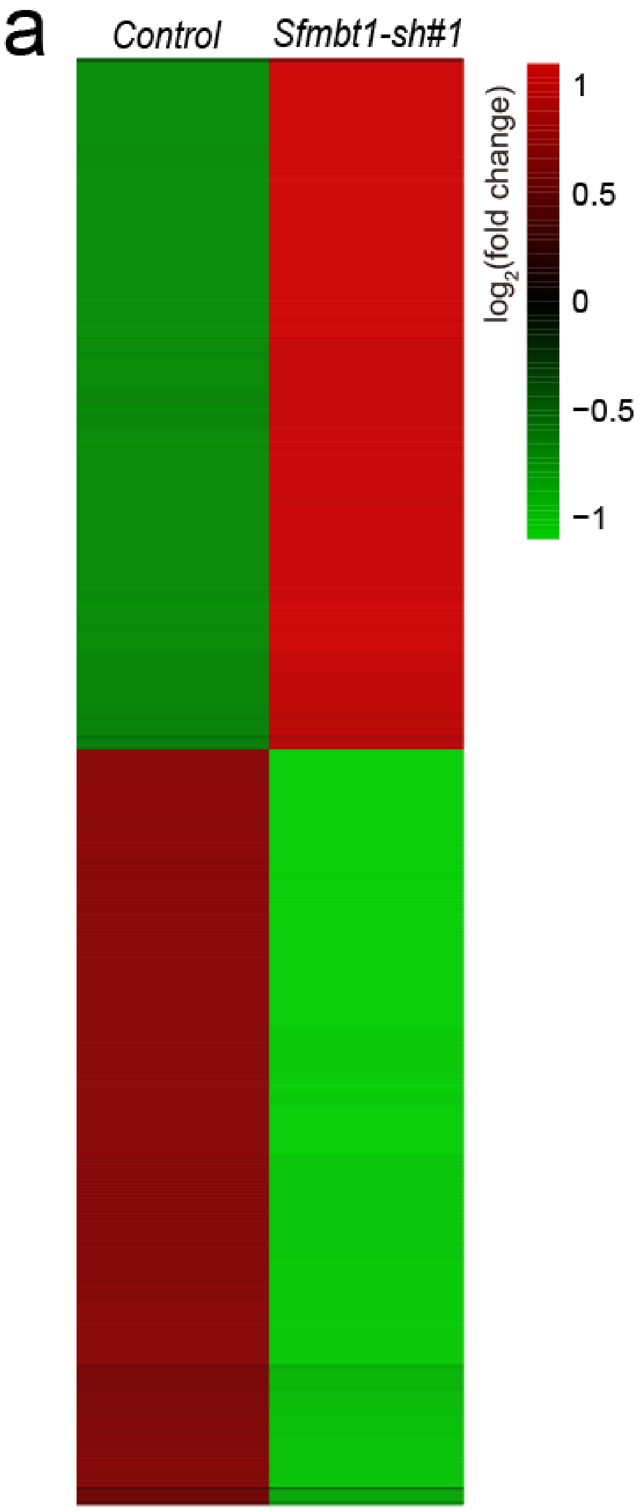

Supplementary Figure 4. Expression heatmap showed the differentially expressed genes (n=3,092, *P*adj<0.01, |Log$_2$(fold change)|<0.5) detected in *Sfmbt1* knocked-down cells (compared with controls).

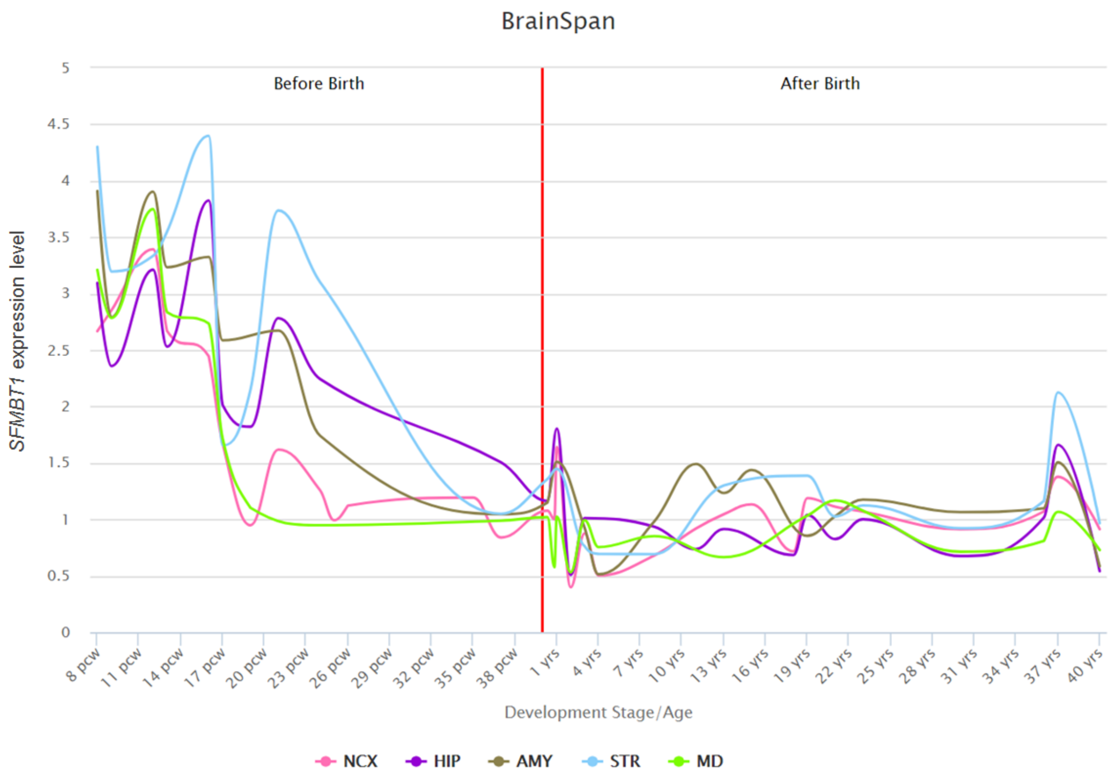

Supplementary Figure 5. Spatio-temporal expression pattern analysis of *SFMBT1* in the developing (prenatal stages) and adulthood brains. *SFMBT1* expression level across different developmental stages (from 8 post-conception weeks (pcw) to 40 years (yrs)) were plotted in five regions (NCX: neocortex, HIP: hippocampus, AMY: amygdaloid complex, STR: striatum and MD: mediodorsal nucleus of thalamus) of the human brain. Data were from the BrainSpan (http://www.brainspan.org/) [9].

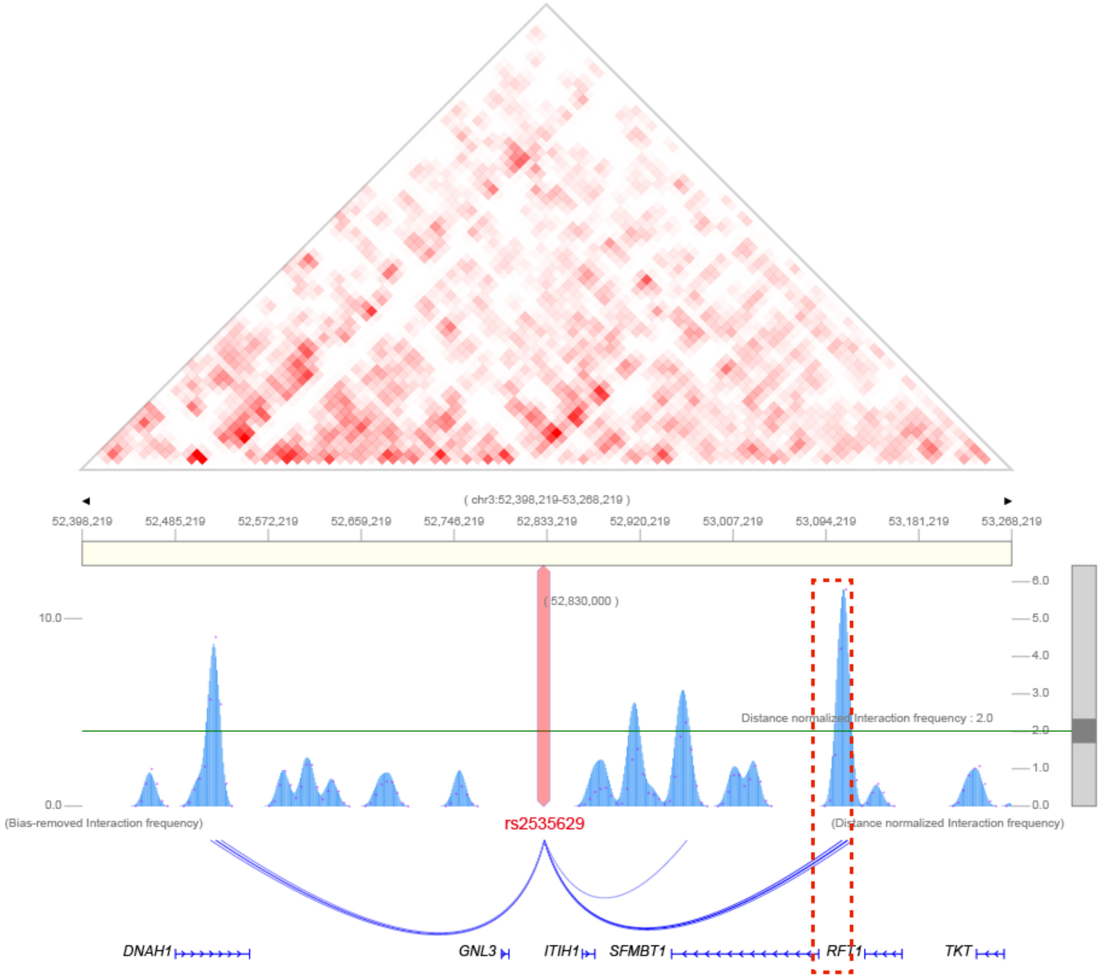

Supplementary Figure 6. rs2535629 was physically interacted with *SFMBT1* (including the promoter regions) in the human dorsolateral prefrontal cortex.

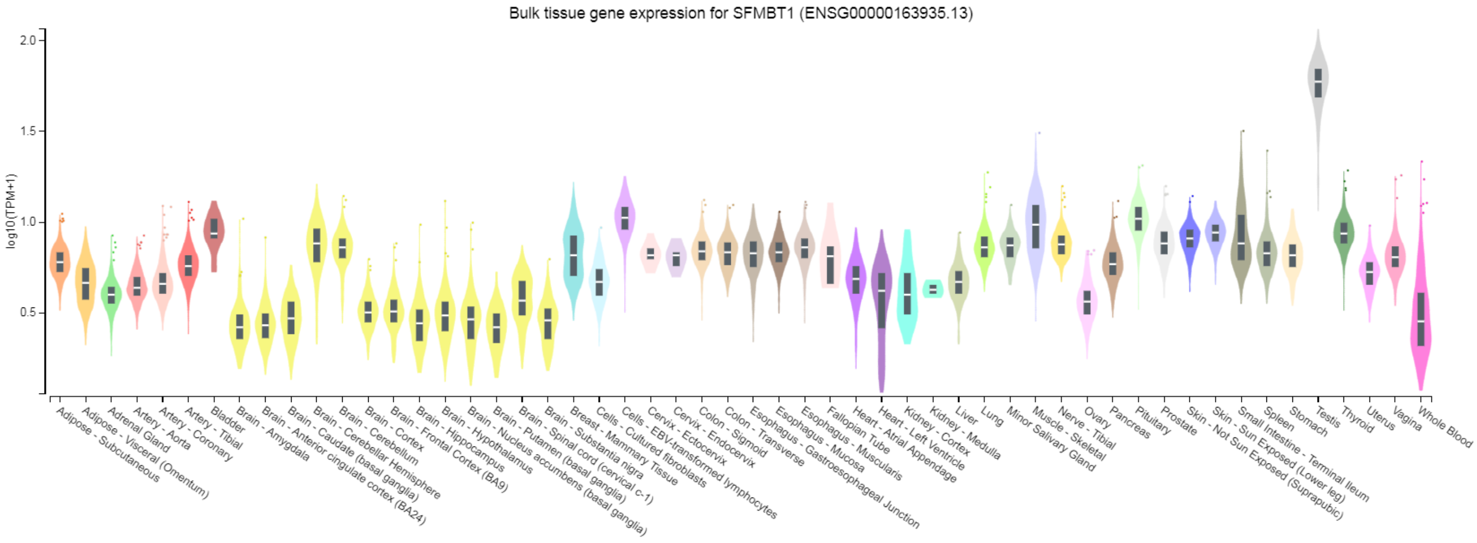

Supplementary Figure 7. *SFMBT1* in different human tissues. Data were from the GTEx website (https://www.gtexportal.org/).

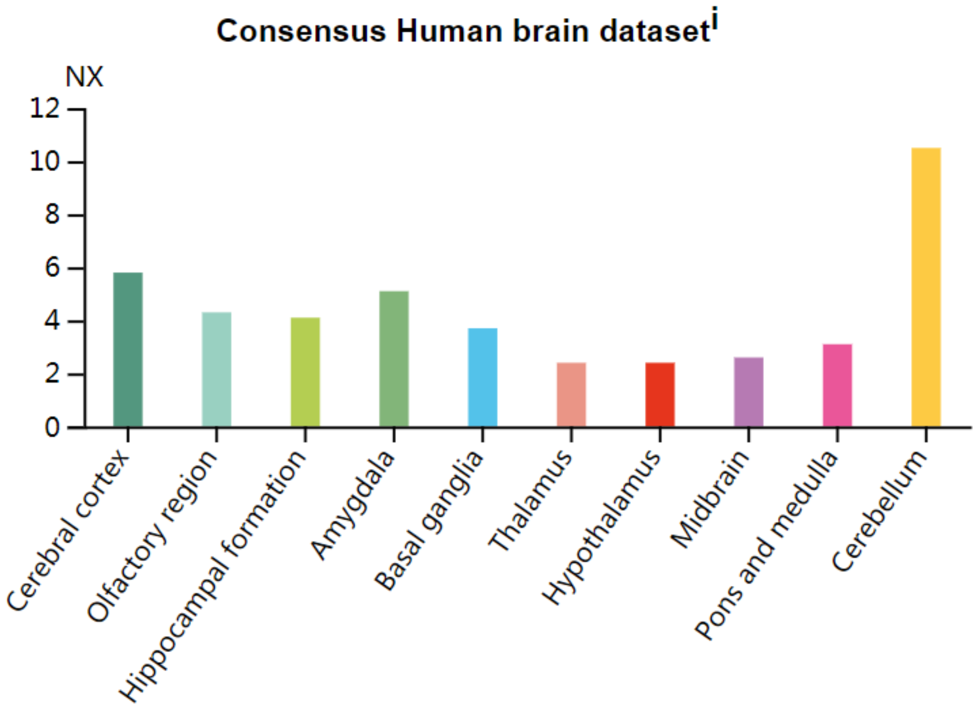

Supplementary Figure 8. *SFMBT1* is widely expressed in the brain. Consensus normalized expression (NX) levels were created for the 10 brain regions by combining the data from two transcriptomics datasets (GTEx and FANTOM5). Color coding is based on brain region and the bar shows the highest expression among the subregions included.

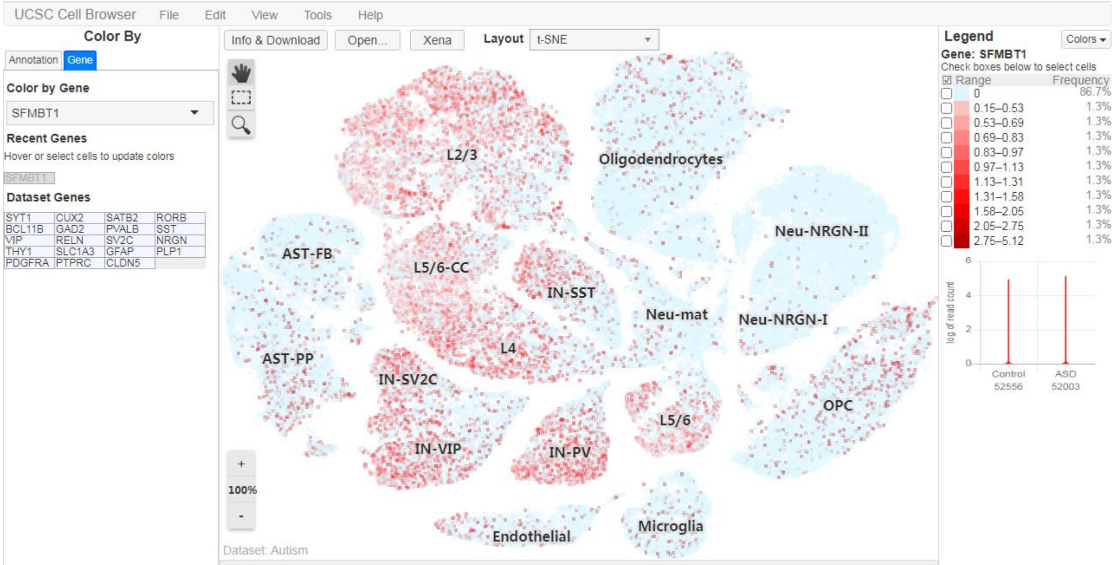

Supplementary Figure 9. Cell-type-specific expression analysis showed that *SFMBT1* expression is relatively high in L2/3 (upper-layer excitatory neurons), L5/6 (deep-layer cortico-cortical excitatory projection neurons), VIP interneurons and parvalbumin interneurons. Data was from the UCSC Cell Browser (https://cells.ucsc.edu/?ds=autism&gene=SFMBT1#).

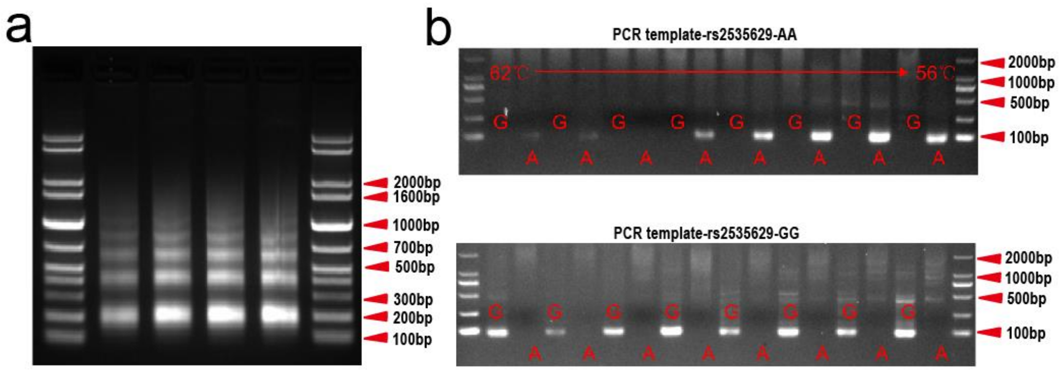

Supplementary Figure 10. Electrophoresis of ChIP samples and primers used for ChIP-AS-qPCR (a) Micrococcal nuclease cleavage of DNA samples into 150-900 bp fragments. **(b)** Primer specificity and temperature gradient assays for ChIP-AS-qPCR.

References

[1] B.S. Runeson, C.L. Rich, *Ann Clin Psychiatry*. **1994**, 6, 181-183.

[2] M.Y. Li, W.Y. Miao, Q.Z. Wu, S.J. He, G. Yan, Y. Yang, J.J. Liu, M.M. Taketo, X. Yu, *Neuron*. **2017**, 94, 1155-1172.e1158.

[3] A. Rodriguez, D.B. Ehlenberger, P.R. Hof, S.L. Wearne, *Nat Protoc*. **2006**, 1, 2152-2161.

[4] D.P. Srivastava, K.M. Woolfrey, P.J.J.o.V.E. Penzes. **2011**. (53):e2794. Doi: 10.3791/2794.

[5] M. Zagrebelsky, A. Holz, G. Dechant, Y.-A. Barde, T. Bonhoeffer, M. Korte, *The Journal of Neuroscience*. **2005**, 25, 9989.

[6] Z.Q. Li, J.H. Chen, H. Yu, L. He, Y.F. Xu, D. Zhang, Q. Yi, C. Li, X. Li, J. Shen, Z. Song, W. Ji, M. Wang, J. Zhou, B. Chen, Y. Liu, J. Wang, P. Wang, P. Yang, Q. Wang, G. Feng, B. Liu, W. Sun, B. Li, G. He, W. Li, C. Wan, Q. Xu, W. Li, Z. Wen, K. Liu, F. Huang, J. Ji, S. Ripke, W. Yue, P.F. Sullivan, M.C. O'Donovan, Y. Shi, *Nat Genet*. **2017**, 49, 1576-1583.

[7] A.F. Pardinas, P. Holmans, A.J. Pocklington, V. Escott-Price, S. Ripke, N. Carrera, S.E. Legge, S. Bishop, D. Cameron, M.L. Hamshere, J. Han, L. Hubbard, A. Lynham, K. Mantripragada, E. Rees, J.H. MacCabe, S.A. McCarroll, B.T. Baune, G. Breen, E.M. Byrne, U. Dannlowski, T.C. Eley, C. Hayward, N.G. Martin, A.M. McIntosh, R. Plomin, D.J. Porteous, N.R. Wray, A. Caballero, D.H. Geschwind, L.M. Huckins, D.M. Ruderfer, E. Santiago, P. Sklar, E.A. Stahl, H. Won, E. Agerbo, T.D. Als, O.A. Andreassen, M. Baekvad-Hansen, P.B. Mortensen, C.B. Pedersen, A.D. Borglum, J. Bybjerg-Grauholm, S. Djurovic, N. Durmishi, M.G. Pedersen, V. Golimbet, J. Grove, D.M. Hougaard, M. Mattheisen, E. Molden, O. Mors, M. Nordentoft, M. Pejovic-Milovancevic, E. Sigurdsson, T. Silagadze, C.S. Hansen, K. Stefansson, H. Stefansson, S. Steinberg, S. Tosato, T. Werge, G. Consortium, C. Consortium, D.A. Collier, D. Rujescu, G. Kirov, M.J. Owen, M.C. O'Donovan, J.T.R. Walters, *Nat Genet*. **2018**, 50, 381-389.

[8] Schizophrenia Working Group of the Psychiatric Genomics Consortium, *Nature*. **2014**, 511, 421-427.

[9] H.J. Kang, Y.I. Kawasawa, F. Cheng, Y. Zhu, X. Xu, M. Li, A.M. Sousa, M. Pletikos, K.A. Meyer, G. Sedmak, T. Guennel, Y. Shin, M.B. Johnson, Z. Krsnik, S. Mayer, S. Fertuzinhos, S. Umlauf, S.N. Lisgo, A. Vortmeyer, D.R. Weinberger, S. Mane, T.M. Hyde, A. Huttner, M. Reimers, J.E. Kleinman, N. Sestan, *Nature*. **2011**, 478, 483-489.
